# Enhancing Biogenic Formic Acid Production in the Modified OxFA Process by Acetonitrile Addition

**DOI:** 10.1002/advs.75647

**Published:** 2026-05-10

**Authors:** Jan‐Dominik H. Krueger, Pegah Saedi, Maximilian J. Poller, Alberto Collauto, Jason P. Hallett, David Robinson, Maxie M. Roessler, Jakob Albert

**Affiliations:** ^1^ Institute of Technical and Macromolecular Chemistry University of Hamburg Hamburg Germany; ^2^ Department of Chemistry and Centre for Pulse EPR Spectroscopy Imperial College London Molecular Sciences Research Hub White City Campus London UK; ^3^ Department of Chemical Engineering Imperial College London London UK; ^4^ Department of Chemistry and Forensics School of Science and Technology Nottingham Trent University Nottingham UK

**Keywords:** acetonitrile, biomass oxidation, DFT calculations, labeling experiments, OxFA process, polyoxometalate, pulse EPR spectroscopy

## Abstract

Developing homogeneously catalyzed, selective biomass transformation techniques toward an industrially viable biomass valorization process is one of the major tasks of a more sustainable chemical industry. Specifically, the production of short‐chain carboxylic acids like formic acid (FA) in the OxFA process is a promising strategy. In this study, we show the beneficial effect of using acetonitrile as a co‐solvent in the modified OxFA process outperforming methanol, demonstrating improved reaction kinetics combined with high selectivity for the HPA‐2 (H_5_PV_2_Mo_10_O_40_) catalyzed oxidation of xylose to FA. Ex situ spectroscopic ^51^V‐NMR as well as optical UV–vis and electrochemical SWV investigations in combination with advanced pulse EPR measurements and DFT calculations clearly reveal the direct interactions of the co‐solvents methanol and acetonitrile with the HPA‐2 catalyst. This leads to improved selectivity for methanol addition whereby acetonitrile addition leads to both enhanced kinetics and improved selectivity on the kinetics of xylose oxidation to FA compared to the classical OxFA process in pure aqueous solution. This study shows interesting new correlations allowing us to further push the limits of the OxFA technology toward higher productivity.

## Graphical abstract

In this study, the beneficial effect of using acetonitrile as a co‐solvent in the modified OxFA process is shown, outperforming methanol, demonstrating improved reaction kinetics combined with high selectivity for the HPA‐2 (H_5_PV_2_Mo_10_O_40_) catalyzed oxidation of xylose to FA.

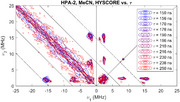

## 1 Introduction

In recent years, the sustainable production of formic acid (FA) from biomass has attracted considerable attention as a promising way to mitigate greenhouse gas emissions and advance the bio‐economy, given its versatile applications as a platform chemical and renewable energy source [1, 2, 3, 4, 5, 6].

Currently, FA is mostly produced via the methanol carbonylation pathway using fossil resources. This process requires harsh conditions and generates toxic by‐products [1, 7, 8]. Traditionally, FA has been used as a reagent in the synthesis of pharmaceuticals, leather, and fine chemicals, and it is important in agriculture due to its use as a preservative and antibacterial agent in livestock feed. Recently, interest in FA has increased due to its potential use as a hydrogen carrier thanks to its chemical and physical properties, not to mention the fact that it is a non‐toxic liquid at room temperature [9]. When hydrogen is required, FA can be catalytically decomposed into H_2_ and CO_2_ under mild reaction conditions [10].

The focus on the sustainable production of FA is increasing, as reducing the environmental footprint is essential for achieving climate goals. This can be accomplished by selecting biomass or carbon dioxide as feedstocks, thereby enabling a circular carbon economy [11, 12, 13]. Due to its various forms and regenerative aspects, biomass is expected to play a significant role. Taking all four generations of biomass into account [14] can ensure a secure and sustainable supply of raw materials compared to finite fossil resources [15, 16]. However, hemicellulosic biomass, a second‐generation biofuel source, is underutilized in chemical processing due to its complex, heterogeneous structure and the subsequent challenges in valorization [17, 18]. Especially xylose, the most abundant sugar in the hemicellulosic fraction of lignocellulosic biomass [19, 20], is a promising substrate for a future bioeconomy as it is mainly transferred into the liquid phase (black liquor) during delignification and therefore underutilized [10].

A recently established method for sustainable FA production is the OxFA process, using polyoxometalate (POM) catalysts for the selective catalytic oxidation of biomass under mild reaction conditions (80°C–120°C), applying water as a solvent and molecular oxygen or compressed air as oxidant [21]. This low‐cost and environmentally friendly process utilizes a vanadium‐substituted HPA‐2 (H_5_PV_2_Mo_10_O_40_) POM catalyst that facilitates oxidative C─C bond cleavage [22, 23, 24]. However, the classical OxFA process in pure aqueous solution suffers from limited carbon efficiency, as around 40% of the carbon initially present in biomass gets lost as CO_2_ [25, 26, 27, 28].

This problem was tackled by different approaches. Several publications deal with process modifications by introducing in situ extraction of FA using a biphasic solvent mixture [29], improving gas entrainment using a Taylor‐flow reactor for more efficient mixing [30, 31, 32] or changing the reactant equivalents [33]. Another possible modification can be made by changing the solvent [34]. Several studies by various groups have shown that using short‐chain alcohols leads to an improved selectivity to FA but comes along with drastically lower reaction kinetics [35, 36] or the formation of undesired side products [37]. Insights into the catalytic behavior of the used POM catalyst have emerged from spectroscopic investigations of glucose transformation reactions with high contents of organic additives (>50%) [37]. Recent in situ investigations have confirmed that the principles of the catalytic mechanism using short‐chain alcohols as co‐solvents are consistent with the OxFA process and revealed the catalytically active structural motif as two adjacent VO_6_ octahedra in the POM structure [38]. Moreover, the redox activity of the active V^V^ species could be modified leading to improved reaction kinetics by the addition of reactive additives like oxalic acid or acetic acid [39].

Until now, various analytical methods, primarily heteronuclear nuclear‐magnetic resonance (NMR) with complementary electron paramagnetic resonance (EPR) and ultraviolet–visible (UV–vis) spectroscopies, have been used to study the vanadium redox chemistry of the POM catalyst in aqueous solution [40, 41, 42, 43, 44]. Another important technique to reveal different catalytic properties of the active vanadium species in solution are electrochemical measurements, as this provides direct insight into changes in oxidation state and electron transfer processes [45, 46, 47]. In particular, multiple redox transitions involving V and molybdenum can be analyzed, and the results can be used to manipulate the catalyst's redox behavior enabling a significant electrocatalytic activity [48].

Moreover, density‐functional theory (DFT) calculations can help to reveal these interactions by showing how solvent molecules influence binding geometries and electronic pathways, as well as interactions between the substrate, solvent, POM catalyst, and other additives [49, 50, 51, 52]. These insights are essential for the understanding of the key POM‐solvent interactions, leading to improved catalytic performance.

Since our previous study showed that the effect of methanol (MeOH) as a co‐solvent dominated over the effect of carboxylic acids as reactive additives [39], we decided to now further explore the potential of co‐solvents. For this purpose, we tested acetonitrile (MeCN) as a representative of a different family (i.e., nitriles) of polar, aprotic molecules. Furthermore, we combined these tests with 2D pulse hyperfine EPR spectroscopy (hyperfine sublevel correlation spectroscopy, HYSCORE), UV–vis‐ and NMR‐spectroscopy, electrochemical analysis, and DFT calculations in an effort to gain a better understanding of the underlying molecular mechanisms. This includes experiments with ^15^N,^13^C‐labeled acetonitrile and ^13^C‐labeled methanol, to elucidate specific catalyst‐solvent interactions.

## 2 Results and Discussion

### 2.1 Influence of Co‐Solvents on the Catalytic Performance of the OxFA Process

**FIGURE 1:**
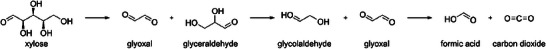
Modified reaction scheme of xylose oxidation in aqueous medium [39].

**FIGURE 2:**
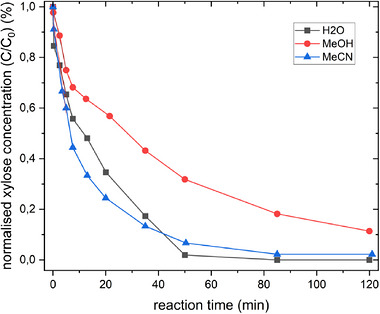
Normalized xylose concentration vs. reaction time for investigated solvents. Reaction conditions: c(H_5_PV_2_Mo_10_O_40_) = 35 mmol/L, c(xylose) = 50 mmol/L, p_O2, start_ = 48 bar, V_stirrer_ = 1500 rpm, solvent composition: 90:10 vol.% H_2_O:MeCN, 90:10 vol.% H_2_O:MeOH, 100% H_2_O;, T = 90°C, t = 120 min, V = 30 mL, Series 5500 reactor.

To evaluate the performance of different co‐solvents in the modified OxFA‐process, we employed a similar model reaction as in our previous studies [39]. Using HPA‐2 as a catalyst, xylose was oxidized to FA (Figure 1) under typical OxFA process conditions (50 bar O_2_, 90°C) in 100% H_2_O or a 9:1 mixture of either H_2_O/MeOH or H_2_O/MeCN (Figure 2). To investigate the effect of a polar aprotic organic solvent without H‐bond formation, we used the simplest cyanide (MeCN) as an alternative additive to the polar protic MeOH [36].

The reaction performed in pure water without additive (classical OxFA‐process) reached full xylose conversion after 50 min. Moreover, the H_2_O/MeOH mixture slowed the reaction rate significantly, leading to a conversion of only 64% at the same reaction time [39]. When the experiment was stopped after 120 min, it had reached a xylose conversion of at least 90%. The acetonitrile addition slightly improved xylose conversion compared to the pure aqueous experiment. The reaction carried out in the H_2_O/MeCN mixture surpassed the purely aqueous system with a conversion of 87% after 35 min of reaction time. At the end of the experiment after 120 min it had also achieved nearly full conversion.

In order to quantify these differences, we used the first five datapoints of each reaction (0–7.5 min) to calculate the initial reaction rates r_obs_ (Equation 4 and Figure S4) and turnover frequencies (TOF) (after 7.5 min, Equation 5), for each solvent mixture (Table 1).

**TABLE 1:** Performance parameters for different solvent mixtures. Reaction conditions: C(H_5_PV_2_Mo_10_O_40_) = 35 mmol/L, c(xylose) = 50 mmol/L, p_O2, start_ = 48 bar, v_stirrer_ = 1500 rpm, solvent composition: 90:10 vol.% H_2_O:MeCN, 90:10 vol.% H_2_O:MeOH, 100% H_2_O; T = 90°C, t = 120 min, V = 30 mL, Series 5500 reactor.

Additive	Reaction rate r_obs_ (mol∙L^−1^∙min^−1^)	Conversion xylose after 120 min (%)	TOF (h^−1^)	Selectivity FA (%)
H_2_O	2.65	100	10.1	44
10 vol.% MeOH	1.89	89	11.0	84
10 vol.% MeCN	3.20	98	19.2	84

The pure aqueous experiment, which is used as a benchmark, shows an initial reaction rate r_obs_ of 2.65 mol L^−1^ min^−1^ and a TOF of 10.1 h^−1^. The fastest experiment using the H_2_O/MeCN mixture shows an improved initial reaction rate r_obs_ of 3.20 mol L^−1^ min^−1^ and a TOF of 19.2 h^−1^, which is significantly faster than the reaction in the H_2_O/MeOH mixture with an initial reaction rate r_obs_ of 1.89 mol L^−1^ min^−1^ and TOF of 11.0 h^−1^. This means that addition of MeCN outperforms both the classical aqueous and the H_2_O/MeOH solvent mixture with respect to reaction kinetics.

**FIGURE 3:**
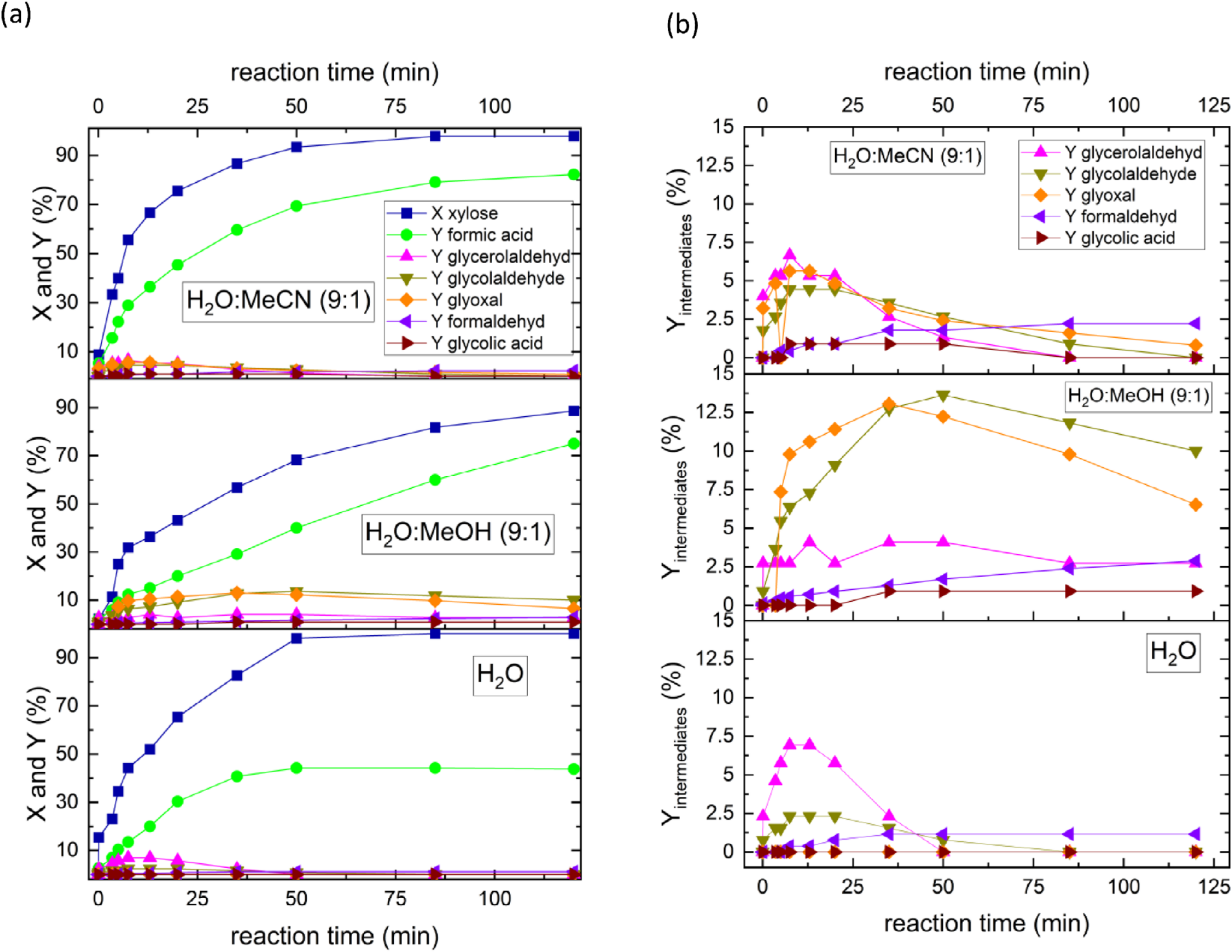
a) Time‐resolved conversion and yield for investigated solvents: aqueous (bottom), methanolic (middle), and acetonitrilic (top) experiment determined by HPLC. B) Zoom‐in for intermediates formation; Reaction conditions: c(H_5_PV_2_Mo_10_O_40_) = 35 mmol/L, c(Xylose) = 50 mmol/L, p_O2, start_ = 48 bar, V_stirrer_ = 1500 rpm, solvent composition: 90:10 vol.% H_2_O:ACN, 90:10 vol.% H_2_O:MeOH, 100% H_2_O; T = 90°C, t = 120 min, V = 30 mL, Series 5500 reactor.

Interestingly, liquid and gas phase analysis revealed the same reaction mechanism for xylose oxidation (see Figure 3 for detailed results) in all tested solvent mixtures via the intermediates glyceraldehyde, glycolaldehyde, glyoxal, and formaldehyde (see Figure S6 for exemplary HPLC chromatogram and Tables S1–S3 for detailed results).

FA yield was increasing all along the reaction coordinate showing continuous conversion of substrate and intermediates with up to full xylose conversion for water and H_2_O/MeCN respectively 90% for H_2_O/MeOH after 120 min reaction time (Figure 3a). A closer look at the different samples over reaction time (Figure 3b) reveals the formation of the intermediates glyceraldehyde, glycolaldehyde, and glyoxal. Glyoxal was not detected in purely aqueous solution, which is unsurprising as previous studies show that it is converted very quickly [39], and therefore does not accumulate in significant quantities. The highest intermediate yield in the pure aqueous solution was achieved for glyceraldehyde (Y = 7%), whereby glycolaldehyde was present in smaller quantities (Y = 2%). Subsequently, all intermediates were converted at a lower rate when methanol was used as a co‐solvent. In the H_2_O/MeOH mixture, glyoxal (Y = 13%) and glycol aldehyde (Y = 14%) were converted more slowly, showing a maximum between 35 and 50 min.

However, in the accelerated reaction in H_2_O/MeCN, glyoxal was formed at an increased rate, leading to the accumulation of a small amount (Y = 4%). Overall, most intermediates were observed in the beginning of the reaction time between 0 and 35 min.

In summary, the evaluation of the intermediate formation confirms that the reaction pathway remains fundamentally the same in each solvent mixture. This was further verified by performing a dedicated lab‐to‐lab comparison in the two different setups applied in the Hallett lab (London), as well as in the Albert lab (Hamburg). For experiments in the Albert lab, CO_2_ yields were determined by GC. For the aqueous reaction after 120 min, a Y_CO2_ of 43.1% was detected, being in line with previous studies. For the experiment using MeOH as a co‐solvent, Y_CO2_ of 1.1 and for the experiment using MeCN a Y_CO2_ of 8.6% was determined. Details can be found in Tables S4–S6 and Figures S3–S5.

### 2.2 Understanding Catalyst‐Solvent Interactions Based on DFT Calculations

**FIGURE 4:**
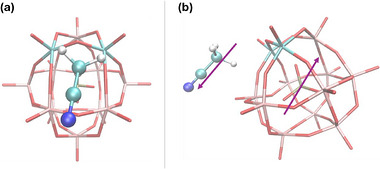
Orientation of MeCN bound to reduced HPA‐2. (a) Orientation viewed from the front. (b) Orientation viewed from the side, with relative orientation of the dipole moments shown as arrows (not to magnitude scale). In the wireframe representation, O atoms are red, Mo atoms are pink and V atoms are cyan. In the CPK representation of MeCN, carbon atoms are cyan, nitrogen atoms are blue and hydrogen atoms are white.

In Figure 4a, the favored binding orientation of MeCN to HPA‐2 in the reduced form is shown (the oxidized form is similar; see Figure S7). In both cases, the favored position is aligned with the vanadium centers, with a parallel orientation of MeCN and the vertical axis through HPA‐2. This matches the favored binding site between HPA‐2 and MeOH in both the oxidized and reduced forms of HPA‐2 (the binding energies can be found in Table S7 and Figure S8). While MeOH and other additives have previously been considered [39] hydrogen‐bonded to the bridging oxygen between the two vanadium centers, MeCN forms a dipole‐dipole interaction, with the dipole moments of MeCN and HPA‐2 aligning anti‐parallel (Figure 4b). Upon aligning with reduced HPA‐2, the electronic distribution within MeCN changes (see Figure S8), but there is no transfer of electron density from the POM to MeCN (or vice versa).

Upon binding of MeCN to reduced HPA‐2 (Figure 4a), a considerable asymmetry in the spin density is seen at the vanadium centers, with more than twice the spin density on one site vs. the other, and further significant spin density seen on any Mo center on the opposite side of the POM. Without additives bound, the spin density of the two vanadium centers is equal, with no appreciable spin density at any of the Mo sites. When either water or MeOH is bound at the vanadium centers, the spin density of the vanadium atoms is equal, again with no significant spin density elsewhere (Table S7) [38, 39]. The binding of MeCN with the reduced form of HPA‐2 is therefore unique in creating a difference in spin density [53], leading to the possibility of the vanadium centers demonstrating different redox ability, and the Mo center with a high spin density also having the ability to participate in redox reactions with MeCN bound [54].

Considering the binding of xylose, we have previously shown that binding at either the Mo–O–V motif or V–O–V motif of HPA‐2 is strongly preferred to the other Mo sites [39]. In both the oxidized and reduced forms of HPA‐2, binding of either MeCN or MeOH is favored over H_2_O. In the case of MeOH, the favored binding site is competitive with xylose for both the oxidized and reduced HPA‐2, which is likely to be responsible for the reduced kinetics in the presence of MeOH [24]. However, with MeCN bound in its favored position (Figure S8), the xylose can bind non‐competitively (with MeCN) to the Mo–O–V motif.

### 2.3 Experimental Evidence for Catalyst‐Solvent Interactions

To validate the theoretical predictions, the catalyst–solvent interactions were investigated using several analytical techniques. First, it was deduced whether changes in solvent composition affect the pH value. No differences were observed, indicating that the HPA‑2 catalyst exhibits the same dissociation behavior in all solvents applied (Table S8). This conclusion was further supported by ^31^P‐ and ^51^V‐NMR spectroscopy, which confirmed the presence of identical catalyst species in each solvent (Figure S9). To gain additional insight into the redox state of the catalyst during the reaction, UV–Vis spectroscopy was subsequently employed. In its oxidized form, [PV^V^_2_Mo_10_O_40_]^5−^ oxidizes the substrate and is thereby reduced to [PV^IV^_2_Mo_10_O_40_]^7−^ [55]. The paramagnetic V^IV^ centers give rise to an intervalence charge‑transfer (IVCT) band at approximately 780 nm [39]. Because diamagnetic V^V^ centers in phosphomolybdates exhibit a dominant ligand‑to‑metal charge‑transfer (LMCT) band at around 330 nm, this lower‑wavelength region was omitted, and only spectra from 500 to 1000 nm were recorded. As expected, the IVCT band at 780 nm was detected and used for relative quantification (Figure S10).

### 2.4 Electrochemical Investigations

In order to further investigate the influence of the co‐solvents MeOH and MeCN on the redox activity of the HPA‐2 catalyst, the electrochemical behavior of HPA‐2 was examined by cyclic voltammetry (CV, Figures S12–S18) and square‐wave voltammetry (SWV, Figure 5) in the three different solvent mixtures.

**FIGURE 5:**
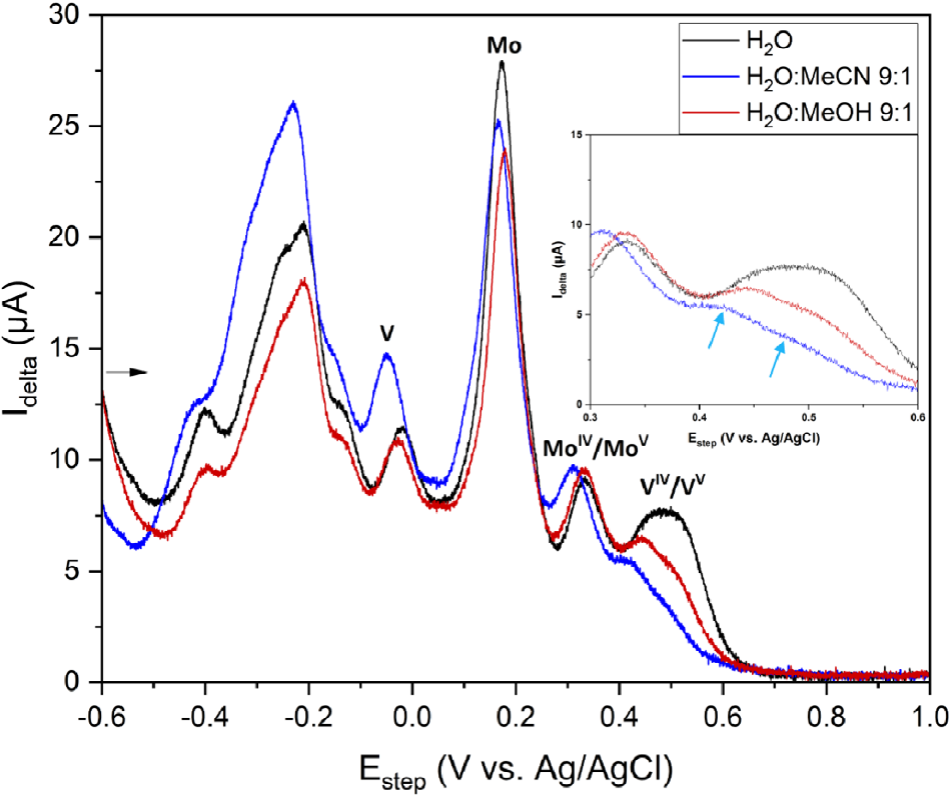
SWV diagram for the HPA‐2 catalyst dissolved in various solvent mixtures. Measurement conditions: Concentration 1 mmol/L, scan rate 5 mV/s, and pH 1 (0.1 M hydrochloric acid was used as supporting electrolyte).

The resulting voltammograms reveal a complex profile, owing to overlapping signals from multiple vanadium and molybdenum species, which complicates the unambiguous assignment of individual redox events. In line with previous reports in aqueous media [21, 56], the first reduction step can be attributed to reduction of V^V^–V^IV^, observed at +464 mV (vs. Ag/AgCl). This is followed by two successive reduction events involving molybdate centers within the POM framework. Additionally, a second vanadium‐centered reduction to V^III^, is observed at –100 mV (vs. Ag/AgCl).

The introduction of 10 vol.% MeOH produced a modest cathodic shift (≈10–20 mV), across the square wave voltammogram most prominently affecting the first reduction event (V^V^–V^IV^). An even more pronounced effect was observed upon addition of MeCN. Not only did this co‐solvent induce a larger cathodic shift, but it also markedly altered the shape of the initial vanadium reduction feature. In water, this process appears as a broad, intense peak (Figure 5, inset), consistent with a simultaneous two‐electron reduction of both vanadium centers. In contrast, MeCN addition diminishes the peak intensity and splits the response into two partially overlapping signals at E = 0.488 V and E = 0.404 V vs. Ag/AgCl (Figure 5, inset: blue arrows). These features either arise from molybdenum‐centered reductions within the framework coinciding with the vanadium‐centered redox process, or from slight differences in reduction potentials between the two vanadium sites, which might be caused by de‐symmetrization upon MeCN coordination, as seen from the DFT calculations.

The more negative oxidation potential of the resulting V^IV^ species in MeCN enables a thermodynamically more favorable reoxidation of the catalyst compared to water without co‐solvents. In combination with the observed V^IV^ concentrations during the reaction, this suggests that improved catalyst re‐oxidation, facilitated by MeCN coordination, plays a key role in steering the selectivity of the overall transformation toward the desired formation of FA.

### 2.5 Investigating Possible Speciation in Different Solvent Mixtures Using CW‐EPR Spectroscopy

EPR spectroscopy is a powerful tool to show paramagnetic V^IV^ species. Therefore, we performed CW‐EPR spectra of the HPA‐2 catalyst in the various solvent mixtures in order to reveal if different V^IV^ species are present. Simulations of the room‐temperature spectra were performed for all the measured samples to extract the corresponding magnetic interaction parameters. In agreement with previous reports on similar systems [37, 57, 58], the V^IV^ center (S = ½) was assumed to be characterized by an axial electron Zeeman (***g***) interaction tensor and a collinear hyperfine (***A***) interaction tensor; the latter takes into account the interaction with the *I* = 7/2 nuclear spin of ^51^V (natural abundance 99.75%). The effect of restricted rotational diffusion was introduced by assuming a spherical diffusion model, characterized by a unique value of the rotational correlation time *τ*_c_; no additional line broadening needed to be introduced. The parameters of the model were optimized using a least‐squares‐based approach (Figures S19–S21). Sample numbering corresponds to the sampling times shown in Tables S10 and S11.

**FIGURE 6:**
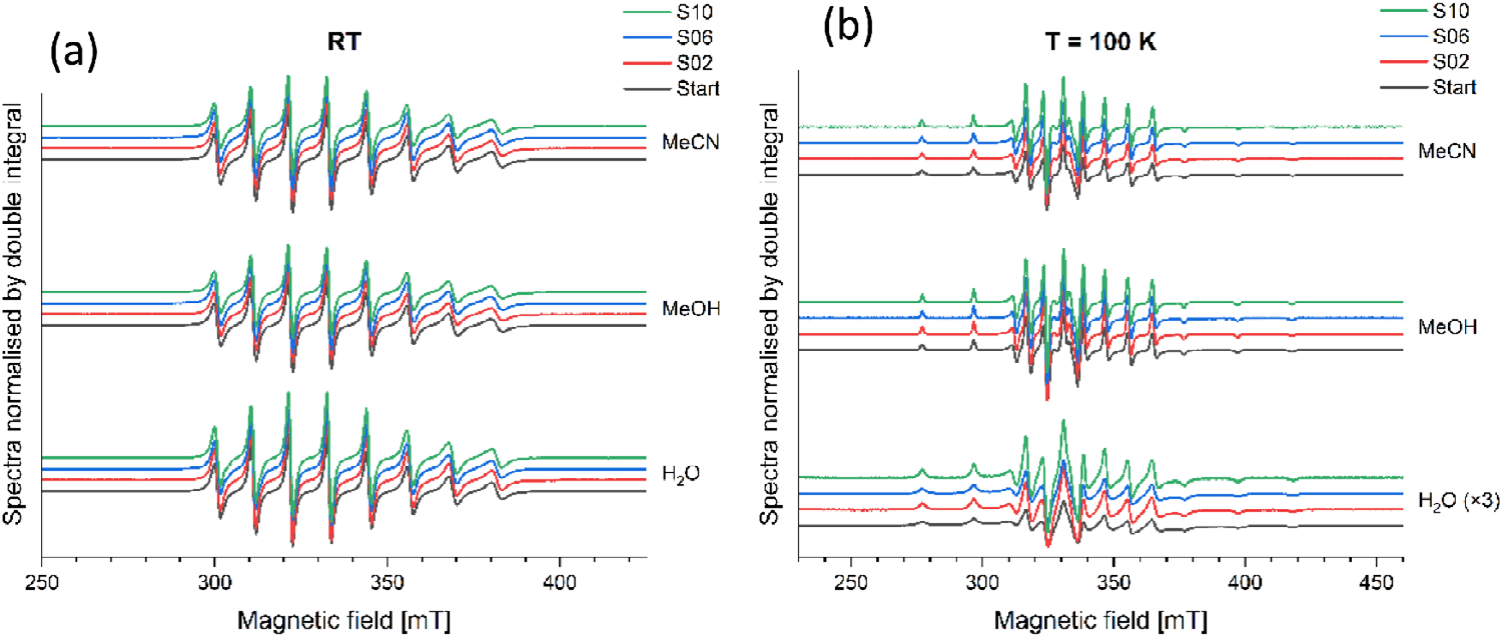
Room‐temperature (left) and 100 K (right) frozen‐matrix CW‐EPR spectra of selected HPA‐2 samples in various solvent mixtures. For improved readability, the 100 K spectra of the samples prepared in the absence of a co‐solvent are multiplied by a factor of 3. As the displayed traces are normalized by the double integral, a weaker signal corresponds to broader lines.

Example X‐band room‐temperature (Figure 6a) and frozen‐matrix (Figure 6b) CW‐EPR spectra only show very little variations between the different solvent mixtures, as also confirmed by the values of the magnetic parameters obtained from the simulations (Table S9). Specifically, differences in *g* values are on the third decimal digit and variations of the hyperfine coupling *A* are in the 1 MHz range. These findings rule out a major impact of the chosen solvent system on the V^IV^ species of HPA‐2.

### 2.6 Comparison of CW‐EPR and UV–Vis Spectral Investigations

**FIGURE 7:**
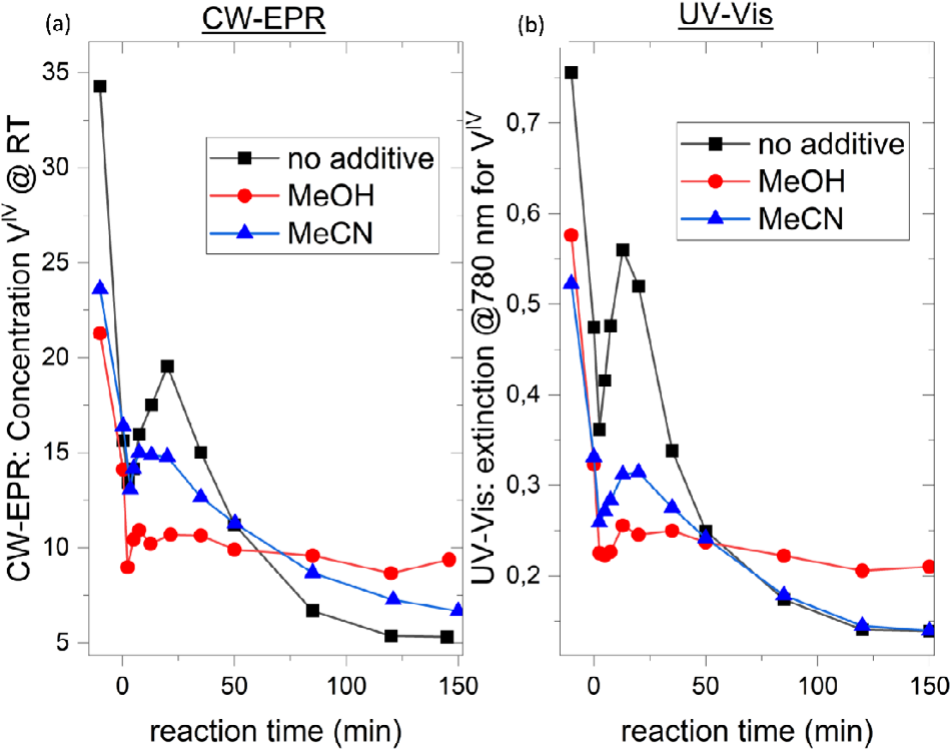
CW‐EPR quantification of V^IV^ content in reaction solutions (a) in comparison with UV–vis absorbance (b) corresponding to V^IV^ content in solution. All spectra measured at room temperature. Measurement conditions: CW‐EPR: microwave power = 20 mW, field modulation amplitude = 0.25 mT at 100 kHz, magnetic field sweep rate = 1.2 mT/s. UV–vis: undiluted samples were measured within 1000 – 500 nm, extinction values were extracted at 780 nm.

As there was no detectable difference of the V^IV^‐species observed before catalysis, we subsequently combined CW‐EPR and UV–vis spectroscopy, to track the oxidation state of the HPA‐2 catalyst over the course of the reaction in the three different solvent mixtures (Figure 7a,b).

Over the course of the reaction, both methods (CW‐EPR, Figure 7a) and UV–vis (Figure 7b) show the same trend for the V^IV^ concentration. At the start of the reaction, the V^IV^ concentration drops sharply. A suitable explanation for this trend could be that during the initial heating phase, the HPA‐2 catalyst is reduced by the substrate, however there is no oxygen entrainment for re‐oxidation yet. At the start of the reaction, the stirrer speed is increased to enable fast oxygen dissolution, whereby the catalyst is re‐oxidized to V^V^ via the formation of an intermediate V^V^‐peroxo species [38, 55]. This in turn results in the substrate oxidation progressing more rapidly, which leads to a subsequent increase in V^IV^ concentration, up to a local maximum. At this point all of the substrate is consumed and the HPA‐2 catalyst is subsequently fully re‐oxidized. This local maximum is least pronounced in H_2_O/MeOH, which fits to the previous observation of slow kinetics (Figure 2) and the presence of intermediates until the end of the course of the reaction (Figure 3). However, it is more pronounced in the aqueous system than in the H_2_O/MeCN mixture, although the addition of MeCN accelerated the overall reaction rate. This indicates that re‐oxidation must be facilitated by the addition of MeCN, since the reduced HPA‐2 catalyst is present in lower concentration despite the reaction progressing faster.

### 2.7 Revealing Catalyst‐Solvent Interactions Using Pulse EPR (HYSCORE) and ^51^V‐NMR Spectroscopy

**FIGURE 8:**
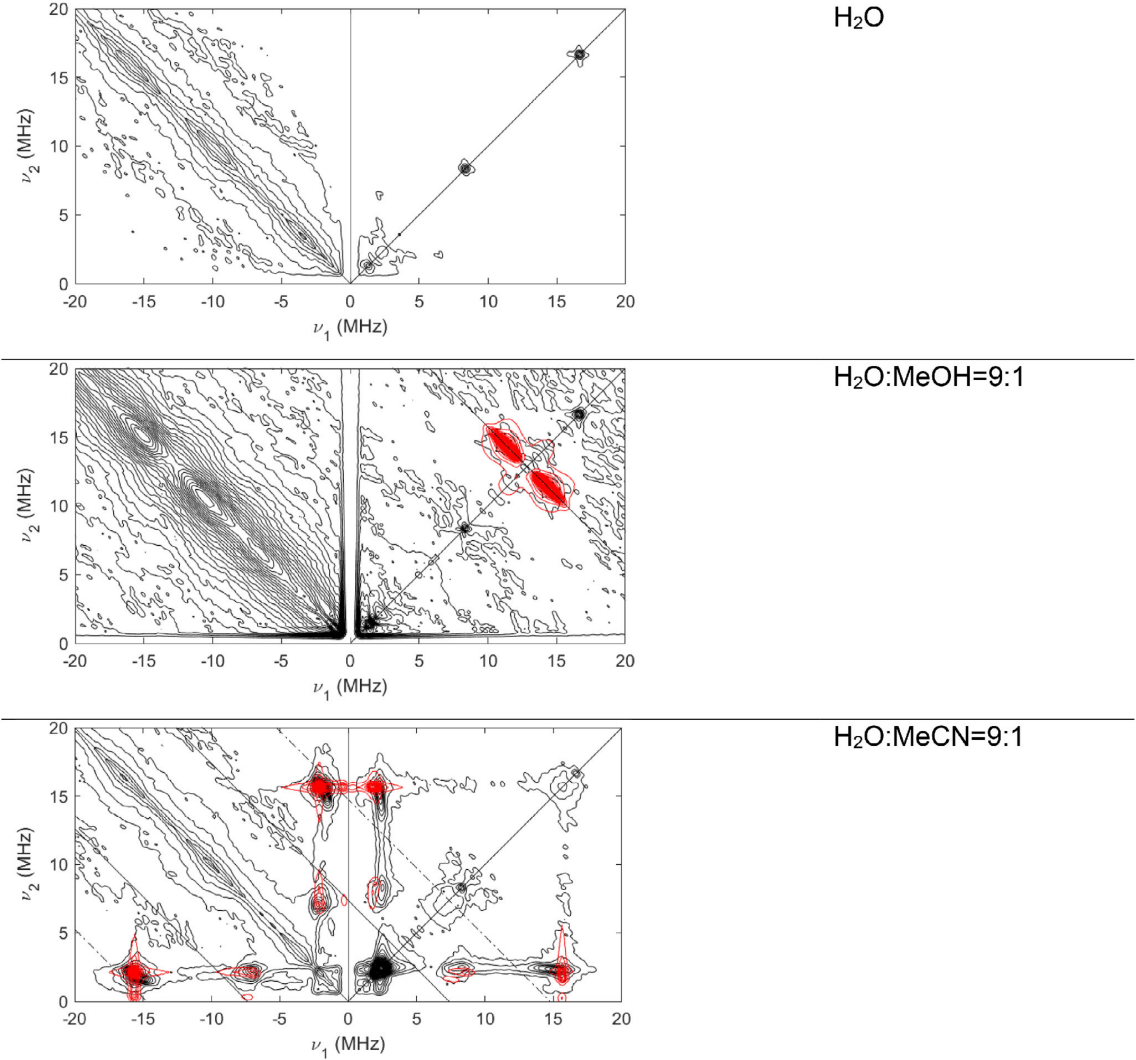
Q‐band (∼34 GHz) HYSCORE spectra of the isotope‐labeled solutions (10 vol.%) with c(HPA‐2) = 35 mmol/L; top: pure water; middle: 9:1 H_2_O:^13^C‐MeOH (black: experimental spectrum; red: simulation, see the text and the Supporting Information for the parameters); bottom: 9:1 H_2_O:MeCN (black: experimental spectrum; red: simulation, see the text and the Supporting Information for the parameters).

**FIGURE 9:**
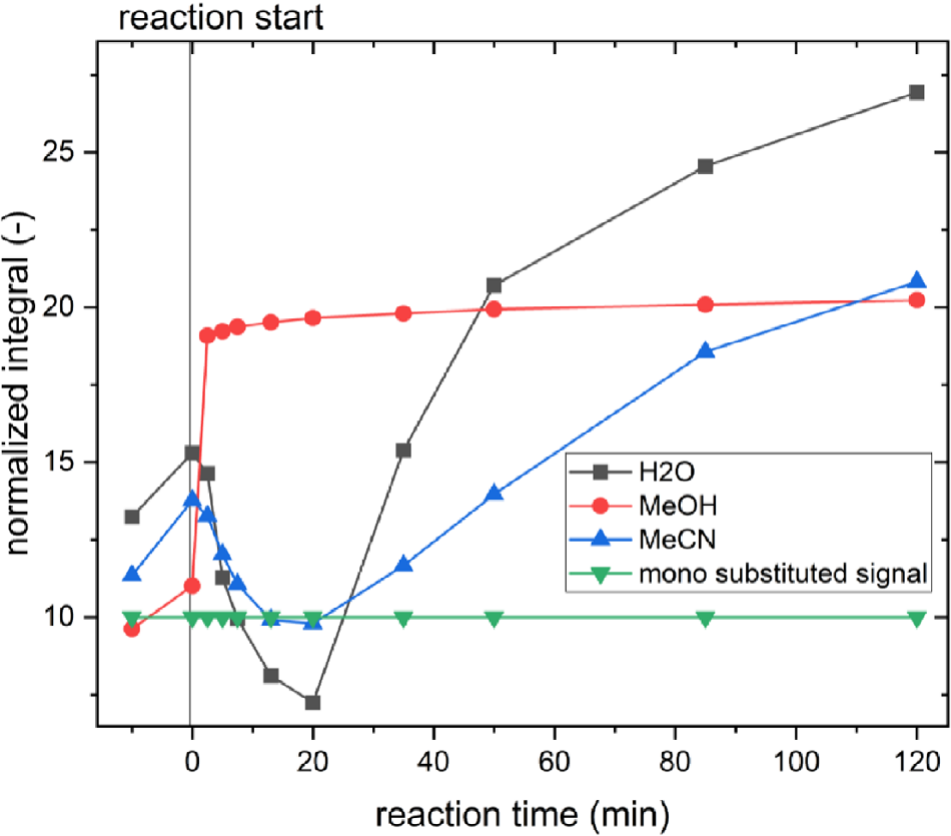
Qualitative/normalized integration of V^v^ from ^51^V‐NMR compared to V^IV^ from CW‐EPR and UV–vis quantification.

In order to observe the interaction of MeCN with the HPA‐2 catalyst directly on a molecular level, we investigated selected samples with 2D Q‐band pulse (Figures S22), HYSCORE pulse EPR (Figure 8) and ^51^V‐NMR spectroscopy (Figure 9).

HYSCORE provides information on the NMR‐active nuclei in the vicinity of the unpaired electron spin (see Figures S24–S31).

In contrast to the HYSCORE spectrum from the sample in pure aqueous solution (Figure 8, top), the spectrum obtained with MeCN (Figure 8, bottom) exhibits a clear set of off‐diagonal cross peaks that must originate from a ^14^N nucleus with *I* = 1. The significant hyperfine coupling (8.2 MHz) and quadrupole splitting (0.6 MHz) obtained from simulations (in red) therefore reveal direct interaction of the V^IV^ spin with ^14^N from MeCN (Figure S24). Importantly, the position of these peaks does not shift over the course of the reaction (Figure S27), suggesting a stable coordination environment during the reaction. The shift of the observed cross‐peaks resulting from isotope labeling with ^15^N (Figure S25) confirms that the observed signals are indeed caused by the interaction between V^IV^ and the nitrogen atom of the co‐solvent. The HYSCORE spectrum obtained from the ^13^C labeled MeOH as a co‐solvent resulted in off‐diagonal peaks (Figure 8, middle) that are not observed in the natural abundance sample. Simulations confirm a bonding interaction with MeOH, with a sizeable through‐bond (2.7 MHz) and through‐space (1.7 MHz) hyperfine interaction to the *I* = ½ ^13^C nucleus (Figure S31).

To complement this investigation of the interaction between MeCN and the reduced HPA‐2 catalyst, we further employed ^51^V‐NMR spectroscopy to study the influence of MeCN on the oxidized form of the HPA‐2 catalyst (Figure S32). As expected, the ^51^V‐NMR spectra show multiple signals, representing the different V‐substitution patterns in HPA‐2. They can be assigned on the basis of a study by Pettersson et al. [59]. The signal at ‐532 ppm is assigned to the mono‐vanadium‐substituted H_4_PVMo_11_O_40_ (HPA‐1) species, which is always present in solutions of V‐substituted phosphomolybdates. Since it has been previously established that this signal is not influenced by the reaction [34], we used it as an internal reference for the chemical shifts. Signals above −534 ppm belong to the α‐1,2, α‐1,5, α‐1,6 isomers, respectively. In our previous work, we identified the α‐1,4 isomer with two adjacent vanadium centres as the catalytically active species [38]. This isomer initially appears at −527 ppm in the aqueous system, at −532 ppm in the H_2_O/MeOH, and at −534 ppm in the H_2_O/MeCN mixtures.

Figure 9 shows normalized integrals of the visible isomers and therefore the V^V^ content in the various reaction solutions. According to a previous study, the monosubstituted HPA‐1 was used as internal reference for normalization. The integrals of the isomers α‐1,4, α‐1,2, α‐1,5, and α‐1,6 were summed and normalized to the corresponding reference signals of HPA‐1 in the different solvent mixtures.

All of the tested solvent mixtures exhibit a higher V^V^ content at the start of the reaction, indicating a slight re‐oxidation of the HPA‐2 catalyst during the heating phase. Subsequently, the aqueous and H_2_O/MeCN solutions show a decrease in V^V^ content until 20 min. Afterward, the V^V^ content rises until the end of the reaction. For the H_2_O/MeOH mixture, only an increase in V^V^ was observed until 10 min; for the remainder of the reaction time, the signal remains constant.

Over the course of the reactions, the signal of the α‐1,4 isomer shifts to higher frequency as the amount of V^IV^ increases, after which it shifts back to lower frequency. This trend matches the concentration of V^IV^ shown in Figure 7 and can be attributed to the influence of paramagnetic species in the sample. It is most pronounced in the purely aqueous experiment and least pronounced in the H_2_O/MeOH mixture. Thereby it confirms our previous conclusions, that the acetonitrile facilitates re‐oxidation of the HPA‐2 catalyst being responsible for the overall improved reaction kinetics.

## 3 Conclusions

In summary, acetonitrile was discovered as a superior additive for the modified OxFA process outperforming methanol and other alcohols mentioned in previous studies. It achieved a very high FA‐selectivity of 84% while exhibiting a higher reaction rate of 3.20 mol L min^−1^ and an outstanding activity with a TOF of 19.2 h^−1^ even compared to the aqueous benchmark system r_obs_ of 2.65 mol L min^−1^ and a TOF of 10.1 h^−1^. By investigating the progression of the V^IV^ concentration using EPR and UV–vis spectroscopies, we could unambiguously confirm the initial substrate oxidation in tandem with the catalyst reduction as the rate‐determining step of the modified OxFA‐process. With HYSCORE measurements we were able to observe the direct interaction of the co‐solvent with the vanadium‐centers of the HPA‐2 catalyst during reaction directly by using ^15^N‐ and ^13^C‐labeled samples. DFT results demonstrated that binding of MeCN to HPA‐2 is favorable, interacting with the vanadium centers and creating an asymmetric spin density distribution. With MeCN bound, xylose is able to bind to the vanadium sites without competing, unlike when MeOH is bound, where xylose and MeOH must compete to bind at the vanadium site. These findings signify a significant step in the improvement of the OxFA process and thereby contribute to a more efficient sustainable production of formic acid.

## 4 Materials and Methods

### 4.1 Materials and Catalyst Synthesis

All chemicals were obtained commercially and used as received without further purification. D(+)‐xylose was supplied by Merck KGaA; the solvents methanol and acetonitrile were supplied by VWR Chemicals and tert‐butanol (99%) as internal standard for NMR measurements was supplied by Grüssing. D_2_O was supplied by Deutero GmbH. ^13^C‐methanol and ^15^N‐^13^C_1_‐acetonitrile for pulse‐EPR measurements were supplied by Sigma Aldrich. Demineralized water (DI) was used as main solvent. For carrying out the catalytic experiments, oxygen (5.0) was bought from Linde AG or BOC.

The vanadium‐substituted HPA‐2 (H_5_PV_2_Mo_10_O_40_) POM catalyst was synthesized according to a new synthesis procedure [55]. The characterization of the catalyst has been carried out using a Fa. Spectro Arcos ICP‐OES device resulting in a P/V/Mo ratio of 1.0/2.0/10.0. The desired Keggin‐structure type was verified by FT‐IR spectroscopy using an IRSpirit‐X equipped with an ATR unit from Shimadzu (Figure S11). Crystal water content was determined to be 15 H_2_O by TGA analysis using a TG 209 F1 Libra from Netsch and a heating ramp of 15 K/min to 350°C.

### 4.2 Experimental Procedure

All kinetic experiments were performed in a three‐fold high‐pressure screening plant (Figure S1) in the Albert lab using a fed‐batch mode setup. Here, three 100 mL autoclaves made from stainless steel (1.4571) can be used independently. All pipes, valves and fittings were made of stainless steel (1.4571). The gaskets used were made of Novaphit MST/XP supplied by Erwin Telle GmbH. In a typical experiment, glass liners were filled with 3.76 g (35 mmol/L) HPA‐2 catalyst, 0.37 g (50 mmol/L) xylose as a substrate and 45 mL of solvent. Here, either 100 vol.% H_2_O or a mixture of 90:10 vol.% H_2_O:MeOH or 90:10 vol.% H_2_O:MeCN were used as solvents. The filled glass liners were inserted into the autoclaves. After closing with the appropriate torque ensuring leak tightness, the autoclaves were purged three times with 35 bar oxygen to ensure a pure oxygen atmosphere. For experiments at a reaction temperature of 90°C, the autoclaves were pressurized to a pre‐pressure of 45 bar. Subsequently, the reaction temperature of 90°C and a stirrer speed of 330 rpm was set. When the inside temperature reached the desired value, the 0‐min sample was drawn from the sampling valve. The stirrer speed was subsequently increased to 1500 rpm. Samples were drawn after shown reaction times and directly put on ice. When the reaction was finished, stirrer speed was decreased to 330 rpm, the heating jackets were taken off and the reactors were cooled with pressurized air. After cooling the reactors to room temperature, samples of the gas phase were taken and subsequently, the autoclaves were vented, and further analysis of the reaction solutions was carried out.

All experiments for spectroscopic investigations were performed in a Parr Series 5500 HP Compact Reactor (Figure S2) in the Hallett lab with a volume of 50 mL made from stainless steel (T316) in a batch mode setup. All pipes, valves and fittings were made of stainless steel (1.4571). In a typical experiment, glass liners were filled with 2.26 g (35 mmol/L) HPA‐2 catalyst, 0.23 g (50 mmol/L) xylose as a substrate and 30 mL of solvent. Here, either 100 vol.% H_2_O or a mixture of 90:10 vol.% H_2_O:MeOH or 90:10 vol.% H_2_O:MeCN were used as solvents. After closing, the autoclaves were purged three times with 35 bar oxygen to ensure a pure atmosphere and put into the heating stand. For experiments at a reaction temperature of 90°C, the autoclaves were pressurized to a pre‐pressure of 42 bar. Subsequently, the reaction temperature of 90°C and a stirrer speed of 330 rpm was set. When the inside temperature reached the desired value, the 0‐min sample was drawn from the sampling valve. The stirrer speed was subsequently increased to 1500 rpm. Samples were drawn after shown reaction times and directly put on ice. When the reaction was finished, stirrer speed was decreased to 0 rpm, and the reactor was taken out of the heating stand. Subsequently, the autoclave was vented, and further analysis of the reaction solutions was prepared.

### 4.3 Analytical Measurements

Ex situ UV–vis spectroscopy was performed using a Cary 60 Spectrophotometer by Agilent. Reaction samples were taken from the reactor and measured non‐diluted in 1 mL cuvettes within 1100–500 nm wavelength range. Afterward, the samples were prepared for NMR‐spectroscopy as well as High Performance Liquid Chromatography (HPLC) and Gas Chromatography coupled with mass spectrometry (GC‐MS) measurements. The catalyst within the reaction solutions was analyzed further by ^51^V‐ and ^31^P‐ NMR spectroscopy using a Bruker Avance III HD 600 MHz spectrometer (base frequency: 600.13 MHz) equipped with a 5 mm BBFO smart probe.

For ex situ EPR spectroscopic investigations, the samples were stored in air‐tight containers and kept on ice till the preparation of the EPR samples, with a maximum delay of 2 h between the sampling and the measurements. For X‐band (≈9.5 GHz) continuous‐wave (CW)‐EPR spectroscopic measurements, the samples from the catalytic experiments were filled into 20 µL glass micropipettes (BLAUBRAND IntraMark, catalogue number 708718). The micropipettes were sealed with Haematocrit sealing compound (BRAND, catalogue number 749510) and loaded into a 2 mm ID, 3 mm OD clear‐fused quartz (CFQ) tube (Wilmad 705‐SQ‐250 M), used as a ‘guiding tube’ to ensure a reproducible sample positioning inside the microwave resonator. The CW‐EPR measurements for each sample were performed both at room temperature and at 100 K using a Bruker EMX X‐band EPR spectrometer equipped with an ER 4119HS high‐sensitivity resonator and an ER 4141VTM nitrogen variable‐temperature unit. To ensure that the measurements at room temperature and 100 K were related to the same state of the catalyst, each sample was flash‐frozen in liquid nitrogen immediately after the acquisition of the room‐temperature spectrum; once all the room‐temperature spectra had been acquired, the 100 K traces were recorded by loading the samples in the pre‐cooled cryostat. The details of the acquisition parameters are reported in section S2.7 of the Supporting Information. The fill height of the micropipettes (62 mm) greatly exceeds the active height of the microwave resonator (45 mm). Therefore, the double integral of the CW‐EPR signal was converted reliably into spin concentrations upon calibrating the response of the spectrometer against a series of copper(II) tetrakis‐imidazole dilutions loaded in the same type of capillaries used for the HPA‐2 solutions. The magnetic field readout was calibrated using a 2,2‐diphenyl‐1‐picrylhydrazyl (DPPH) powder sample as a standard (*g* = 2.0036) to enable an accurate determination of *g* values [57].

For pulse EPR measurements, the samples were filled into bottom‐sealed borosilicate capillary tubes (1.15 mm ID, 1.55 mm OD; Hirschmann, catalogue number 9211590) and subsequently frozen and stored in liquid nitrogen. Measurements were performed at Q‐band frequencies (≈34 GHz, 1.2 T) using a Bruker Elexsys E580 spectrometer equipped with an EN 5107D2 microwave resonator and a 300 W traveling‐wave tube amplifier (Applied Systems Engineering Inc. model 177Ka). The sample temperature was set using a closed‐circuit cryostat (Cryogenic Ltd.) controlled by a Cernox sensor and a Model 350 temperature controller (Lake Shore Cryotronics, Inc.). Acquisition parameters are reported in section S2.7 in the Supporting Information.

All electrochemical measurements were performed in aqueous hydrochloric acid solution at pH 1, with an analyte concentration of 1 mmol/L, using a BioLogic SP‐150e potentiostat (VMP3B‐5 chassis), for details see section S2.2 in the Supporting Information. Prior to each experiment, the electrolyte solution was thoroughly purged with nitrogen gas to remove dissolved oxygen. The electrochemical setup consisted of a 10 mL cell equipped with a conventional three‐electrode configuration: a glassy carbon working electrode (3 mm diameter), a platinum wire counter electrode, and an Ag/AgCl reference electrode. Prior to each measurement in a new solvent mixture, the working electrode was regenerated using standard procedures, including rinsing with water, polishing with 0.05 µm alumina slurry on polishing pads, subsequent washing with Milli‐Q water, and drying.

Cyclic voltammetry (CV) was recorded over a potential window from –0.6 V to +1.0 V (vs. Ag/AgCl) at a scan rate of 100 mV/s for three consecutive scans. Square‐wave voltammetry (SWV) was carried out over the same potential window with instrument settings of PH = 20 mV, PW = 40 ms, and SH = 0.4 mV, corresponding to a modulation amplitude of 20 mV, a frequency of 12.5 Hz, and a scan rate of 5 mV/s. These electrochemical parameters were selected as a compromise between spectral resolution and measurement sensitivity, ensuring reliable detection of overlapping redox events (see section S2.2 and Figures S4–S10).

Careful investigation and measurement optimization were performed in pure aqueous solution (+HCl as supporting electrolyte, see section S2.3 in the Supporting Information) before exploring the effects of solvent composition.

### 4.4 Determination of Quantitative Reaction Parameters

All reaction products were quantitatively determined by HPLC, NMR, and GC analysis. Liquid phase analysis was carried out using HPLC and ^1^H‐NMR spectroscopy. An exemplary HPLC‐Chromatogram can be found in Figure S6. For NMR‐analysis, 500 µL reaction solutions were mixed with 75 µL D_2_O – 10 wt.% t‐BuOH solution. The conversion of substrates and yields of all liquid products were determined by means of HPLC measurements using a Nexera‐40 HPLC from Shimadzu equipped with a 300 × 8 mm organic acid column from CS‐Chromatographie GmbH and a refractive index detector (RID). 4 mmol of an aqueous sulfuric acid solution was used as eluent and the samples were filtrated before analysis through a syringe filter (45 µm). The yields of formic acid and the corresponding esters (in methanol) were quantified by ^1^H‐NMR using a Bruker Avance III HD 600 MHz spectrometer. The determination of the gaseous by‐products CO_2_ and CO was done by means of GC analysis using a Varian GC 450 equipped with a 2 m × 0.75 mm ID ShinCarbon ST column and calculated as n(CO_2_ resp. CO)/n(C‐atoms substrate). No other gaseous products could be detected by the used GC.

Xylose conversion *X*_xylose_ was determined from the HPLC results of the liquid phase according to Equation (1).

(1) $$X_{\mathrm{xylose}}\left(\%\right)=\left(\frac{n_{\mathrm{xylose},0}-n_{\mathrm{xylose}}}{n_{\mathrm{xylose},0}}\right)\cdot 100=\left(\frac{c_{\mathrm{xylose},0}-c_{\mathrm{xylose}}}{c_{\mathrm{xylose},0}}\right)\cdot 100$$

In Equation (1), *n*_xylose, 0_ is the amount and *c*_xylose, 0_ the concentration of xylose at time zero before the start of the reaction and *n*_xylose_ is the amount and *c*_xylose_ the concentration of xylose at the respective time during or after the reaction.

With the determined amount of substance of the gas phase and the measured amount of substance of the liquid phases by HPLC, the respective yield *Y*_i_ can be determined according to Equation (2).

(2) $$\begin{array}{ccc} Y_{i}\left[\%\right] & & =\left(\frac{n_{i}\cdot \Sigma C-\mathrm{Atom}s_{i}}{n_{\mathrm{xylose},0}\cdot \Sigma C-\mathrm{Atom}s_{\mathrm{xylose}}}\right)\cdot 100\\ & & =\left(\frac{c_{i}\cdot \Sigma C-\mathrm{Atom}s_{i}}{c_{\mathrm{xylose},0}\cdot \Sigma C-\mathrm{Atom}s_{\mathrm{xylose}}}\right)\cdot 100 \end{array}$$

In Equation (2), ∑C‐Atoms_i_ is the sum of the C‐atoms of product i, ∑C‐Atoms_xylose_ is the sum of the C‐atoms of xylose and *n*_i_ is the amount and *c*_i_ the concentration of product i.

The selectivity *S*_i_ of each product i is determined from the ratio of the yield *Y*_i_ of product i to the conversion of xylose *X*_xylose_. This is given by Equation (3).

(3) $$S_{i}\;\left[\%\right]=\left(\frac{Y_{i}}{X_{\mathrm{xylose}}}\right)\cdot 100$$

As shown in the previous literature, observed reaction rates r_obs_ can be calculated by Equation (4) [39].

(4) $$r_{\mathrm{obs}}=k_{\mathrm{obs}}\cdot c_{\mathrm{xylose}}^{a}$$

The value k_obs_ is the slope of the linear fit from the normalized xylose concentration vs. time plots and c_xylose_ the initial substrate concentration.

The turnover frequency (TOF) was calculated by Equation (5).

(5) $$\mathrm{TOF}=\frac{\left(\frac{n_{\mathrm{product}}}{n_{\mathrm{active}\mathrm{metal}}}\right)}{t}$$

As we have shown in a previous publication [39], two adjacent vanadium‐containing octahedra are needed for the catalytic activity, *n*_active metal_ is equal to *n*_catalyst_ in this publication.

### 4.5 Computational Details for DFT Calculations

Geometry optimizations of HPA‐2 with MeCN binding to different positions of HPA‐2 (see section S4 in the Supporting Information) were performed using the r2SCAN‐3c functional, using the conductor‐like polarizable continuum model (C‐PCM) implicit solvent model to account for solvation. The nature of the stationary points was confirmed using harmonic vibrational frequency analysis. Binding energies were calculated as the difference in (Gibbs) energy between the bound complex and a supermolecule calculation, in which HPA‐2 and MeCN were separated by at least 10 Å. For supermolecule calculations, imaginary frequencies smaller than −*i*ω  =  20 cm^−1^ were neglected, while for the bound complexes, minima corresponded to those with zero imaginary frequencies. HPA‐2 was considered in both the fully oxidized form (with all Mo atoms having a +6 charge, and V having a +5 charge) and the reduced form, in which each of the V atoms had a charge of +4. Stability analysis of the resulting open‐shell SCF solutions was performed to ensure a stable (broken symmetry) solution. All calculations were performed with Orca 6.

## Author Contributions

J.D.K. performed the catalytic experiments as well as the UV–vis and NMR measurements and wrote the initial draft. P.S. carried out the electrochemical measurements. M.J.P. corrected the draft and supervised the NMR and UV–vis experiments. A.C. performed the EPR measurements. J.P.H. supervised the catalytic experiments in the Hallett lab and reviewed the draft. D.R. performed the DFT calculations and reviewed the draft. M.M.R. supervised the EPR measurements and reviewed the draft. J.A. acquired funding, supervised and coordinated the whole project, and reviewed the draft.

## Conflicts of Interest

The authors declare no conflicts of interest.

## Supporting information




**Supporting File**: advs75647‐sup‐0001‐SuppMat.docx.

## Data Availability

The data that support the findings of this study are openly available in Zenodo at https://10.5281/zenodo.17661358, reference number 10.5281/zenodo.17661358.
