# Supplementary material for: Enhancing Biogenic Formic Acid Production in the Modified OxFA Process by Acetonitrile Addition
Source: Adv Sci (Weinh). 2026 May 10;13(43):e75647. doi: 10.1002/advs.75647 (PMC13335955; doi:10.1002/advs.75647)
Supplement: Supplementary file 1 — Supporting File: advs75647‐sup‐0001‐SuppMat.docx. [file ADVS-13-e75647-s001.docx]

**Enhancing biogenic formic acid production in the modified OxFA process by acetonitrile addition**

*Jan-Dominik H. Krueger^1^, Pegah Saedi^1^, Maximilian J. Poller^1^, Alberto Collauto^2^, Jason Hallett^3^, David Robinson^4^, Maxie M. Roessler^2^, Jakob Albert*^1^*

^1^ Institute of Technical and Macromolecular Chemistry, Universität Hamburg, Bundesstrasse 45, 20146 Hamburg, Germany

^2^ Department of Chemistry and Centre for Pulse EPR Spectroscopy, Imperial College London, Molecular Sciences Research Hub, White City Campus, London W12 0BZ, United Kingdom

^3^ Department of Chemical Engineering, Imperial College London, Bone Building, South Kensington Campus, United Kingdom

^4^ Department of Chemistry and Forensics, School of Science and Technology, Nottingham Trent University, Clifton Lane, Nottingham, NG11 8NS, United Kingdom

[jakob.albert@uni-hamburg.de](mailto:jakob.albert@uni-hamburg.de)

(This supporting information contains 11 tables and 32 figures on 31 pages.)

Contents

[**1.** **Reactor setup** 3](#_Toc226816160)

[1.1 3-fold screening plant (Albert lab) 3](#_Toc226816161)

[1.2 Parr Series 5500 HP Compact Reactor (Hallet lab) 3](#_Toc226816162)

[**2** **Additional information for catalytic reactions** 4](#_Toc226816163)

[2.1 Full results for reactions in Series 5500 HP Compact reactor (Hallet lab at ICL) 4](#_Toc226816164)

[Catalytic results: pure aqueous experiment 4](#_Toc226816165)

[Catalytic results: MeOH influenced reaction 5](#_Toc226816166)

[Catalytic results: MeCN influenced reaction 5](#_Toc226816167)

[Pressure-Temperature-Time profile 6](#_Toc226816168)

[Determination of observed reaction rates r_obs_ 6](#_Toc226816169)

[Exemplary HPLC Chromatogram 7](#_Toc226816170)

[2.2 Full results for reactions in 3-fold screening plant (Albert lab at UHH) 8](#_Toc226816171)

[Catalytic results: pure aqueous experiment 8](#_Toc226816172)

[Catalytic results: MeOH influenced reaction 9](#_Toc226816173)

[Catalytic results: MeCN influenced reaction 10](#_Toc226816174)

[**3.** **Additional computational results** 11](#_Toc226816175)

[3.1 Binding positions to the POM 11](#_Toc226816176)

[3.2 Binding energies and orientations 12](#_Toc226816177)

[**4. Spectroscopic and electrochemical investigation of HPA-2** 13](#_Toc226816178)

[4.1 Preparation of catalyst solution and pH values for spectroscopic analysis 13](#_Toc226816179)

[4.2 NMR Spectroscopy 13](#_Toc226816180)

[4.3 UV-Vis Spectroscopy 14](#_Toc226816181)

[4.4 FT-IR Spectroscopy (solid state) 14](#_Toc226816182)

[4.5 Electrochemical measurements 15](#_Toc226816183)

[4.6 EPR Spectroscopy 18](#_Toc226816184)

[General considerations 18](#_Toc226816185)

[X-band continuous-wave EPR spectroscopy 19](#_Toc226816186)

[Q-band pulse EPR spectroscopy 22](#_Toc226816187)

[HYSCORE experiments on the H_2_O:MeCN samples 24](#_Toc226816188)

[HYSCORE experiments on the H_2_O:MeOH samples 28](#_Toc226816189)

[4.7 Additional results of spectroscopic measurements of reaction samples 30](#_Toc226816190)

[**5.** **References** 36](#_Toc226816191)

# **1. Reactor setup**

## 1.1 3-fold screening plant (Albert lab)


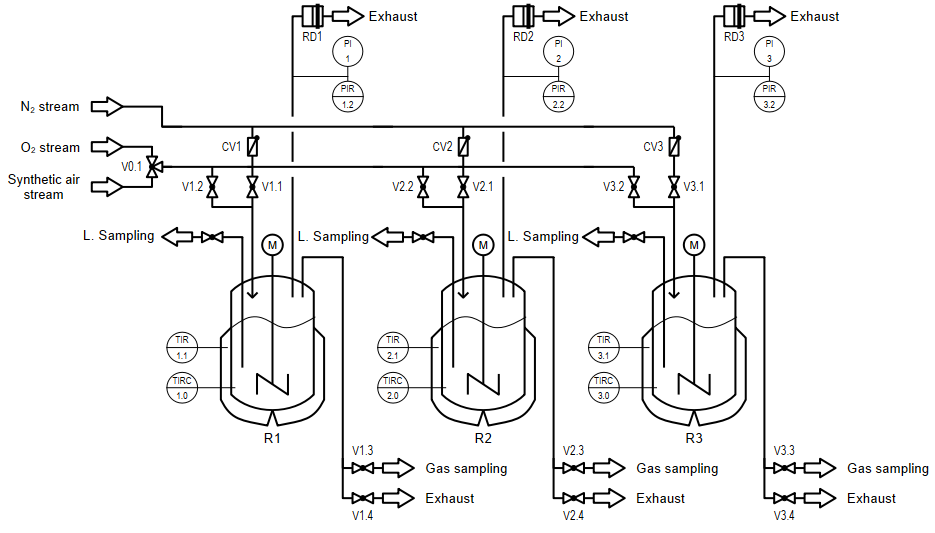


Figure S1: PID scheme of used 3-fold screening plant.

## 1.2 Parr Series 5500 HP Compact Reactor (Hallet lab)


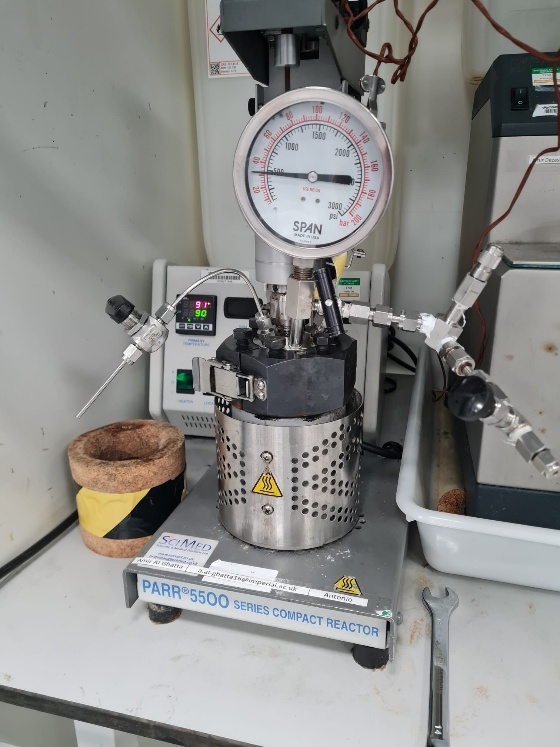


Figure S2: Picture of 50 mL Compact Reactor set-up used in this study.

# **2 Additional information for catalytic reactions**

Furthermore, we assessed the stability of the additives by HPLC and found that approx. 13 % of the respective additive was lost during the reaction. While MeOH most likely decomposed to FA and CO_2_, in the case of MeCN we think of a decomposition to N_2_ as shown for the ECODS process.^1^

## 2.1 Full results for reactions in Series 5500 HP Compact reactor (Hallet lab at ICL)

### Catalytic results: pure aqueous experiment

Table S1: Conversion and yield for non-additive experiment. Reaction conditions: c(H_5_PV_2_Mo_10_O_40_) = 35 mmol/L, c(Xylose) = 50 mmol/L, p_O2, start_ = 46 bar, V_stirrer_ = 1500 rpm (standard Parr impeller), solvent composition: 100 % H_2_O; T = 90 °C, t = 120 min, V = 30 mL.

| **Reaction time (min)** | **X_xylose_ (%)** | **Y_Formic acid_ (%)** | **Y_Glyceraldehyde_ (%)** | **Y_Glycolaldehyde_ (%)** | **Y_Glyoxal_ (%)** | **Y_Formaldehyde_ (%)** |
| --- | --- | --- | --- | --- | --- | --- |
| **0** | 0.00 | 0.00 | 0.00 | 0.00 | 0.00 | 0.00 |
| **0.1** | 15.38 | 2.69 | 2.31 | 0.77 | 0.00 | 0.00 |
| **3.5** | 23.08 | 6.92 | 4.61 | 1.54 | 0.00 | 0.00 |
| **5** | 34.62 | 10.38 | 5.77 | 1.54 | 0.00 | 0.00 |
| **7.5** | 44.23 | 13.46 | 6.92 | 2.31 | 0.00 | 0.38 |
| **13** | 51.92 | 20.00 | 6.92 | 2.31 | 0.00 | 0.38 |
| **20** | 65.38 | 30.38 | 5.77 | 2.31 | 0.00 | 0.77 |
| **35** | 82.69 | 40.77 | 2.31 | 1.54 | 0.00 | 1.15 |
| **50** | 98.08 | 44.23 | 0.00 | 0.77 | 0.00 | 1.15 |
| **85** | 100.00 | 44.23 | 0.00 | 0.00 | 0.00 | 1.15 |
| **121** | 100.00 | 43.85 | 0.00 | 0.00 | 0.00 | 1.15 |

### Catalytic results: MeOH influenced reaction

Methyl formate is cleaved into methanol and formic acid during acidic HPLC measurement.

Table S2: Conversion and yield for methanolic influenced experiment. Reaction conditions: c(H_5_PV_2_Mo_10_O_40_) = 35 mmol/L, c(Xylose) = 50 mmol/L, p_O2, start_ = 46 bar, V_stirrer_ = 1500 rpm (standard Parr impeller), solvent composition: 90:10 % H_2_O:MeOH; T = 90 °C, t = 120 min, V = 30 mL.

| **Reaction time (min)** | **X_xylose_ (%)** | **Y_Formic acid_ (%)** | **Y_Glyceraldehyde_ (%)** | **Y_Glycolaldehyde_ (%)** | **Y_Glyoxal_ (%)** | **Y_Formaldehyde_ (%)** |
| --- | --- | --- | --- | --- | --- | --- |
| **0** | 0.00 | 0.00 | 0.00 | 0.00 | 0.00 | 0.00 |
| **0.1** | 2.27 | 2.27 | 2.73 | 0.91 | 0.00 | 0.12 |
| **3.5** | 11.36 | 5.91 | 2.73 | 3.64 | 0.00 | 0.29 |
| **5** | 25.00 | 9.09 | 2.73 | 5.45 | 7.34 | 0.45 |
| **7.5** | 31.82 | 12.27 | 2.73 | 6.36 | 9.79 | 0.58 |
| **13** | 36.36 | 15.00 | 4.09 | 7.27 | 10.60 | 0.70 |
| **20** | 43.18 | 20.00 | 2.73 | 9.09 | 11.42 | 0.90 |
| **35** | 56.82 | 29.09 | 4.09 | 12.73 | 13.05 | 1.27 |
| **50** | 68.18 | 40.00 | 4.09 | 13.64 | 12.24 | 1.69 |
| **85** | 81.82 | 60.00 | 2.73 | 11.82 | 9.79 | 2.38 |
| **121** | 88.64 | 75.00 | 2.73 | 10.00 | 6.53 | 2.88 |

### Catalytic results: MeCN influenced reaction

Table S3: Conversion and yield for acetonitrile influenced experiment. Reaction conditions: c(H_5_PV_2_Mo_10_O_40_) = 35 mmol/L, c(Xylose) = 50 mmol/L, p_O2, start_ = 46 bar, V_stirrer_ = 1500 rpm (standard Parr impeller), solvent composition: 90:10 % H_2_O:MeCN; T = 90 °C, t = 120 min, V = 30 mL.

| **Reaction time (min)** | **X_xylose_ (%)** | **Y_Formic acid_ (%)** | **Y_Glyceraldehyde_ (%)** | **Y_Glycolaldehyde_ (%)** | **Y_Glyoxal_ (%)** | **Y_Formaldehyde_ (%)** |
| --- | --- | --- | --- | --- | --- | --- |
| **0** | 0.00 | 0.00 | 0.00 | 0.00 | 0.00 | 0.00 |
| **0.1** | 8.89 | 5.33 | 4.00 | 1.78 | 3.21 | 0.00 |
| **3.5** | 33.33 | 15.56 | 5.33 | 2.67 | 4.82 | 0.00 |
| **5** | 40.00 | 22.22 | 5.33 | 3.56 | 0.00 | 0.44 |
| **7.5** | 55.56 | 28.89 | 6.67 | 4.44 | 5.62 | 0.44 |
| **13** | 66.67 | 36.44 | 5.33 | 4.44 | 5.62 | 0.89 |
| **20** | 75.56 | 45.33 | 5.33 | 4.44 | 4.82 | 0.89 |
| **35** | 86.67 | 59.56 | 2.67 | 3.56 | 3.21 | 1.78 |
| **50** | 93.33 | 69.33 | 1.33 | 2.67 | 2.41 | 1.78 |
| **85** | 97.78 | 79.11 | 0.00 | 0.89 | 1.61 | 2.22 |
| **121** | 97.78 | 82.22 | 0.00 | 0.00 | 0.80 | 2.22 |

### Pressure-Temperature-Time profile


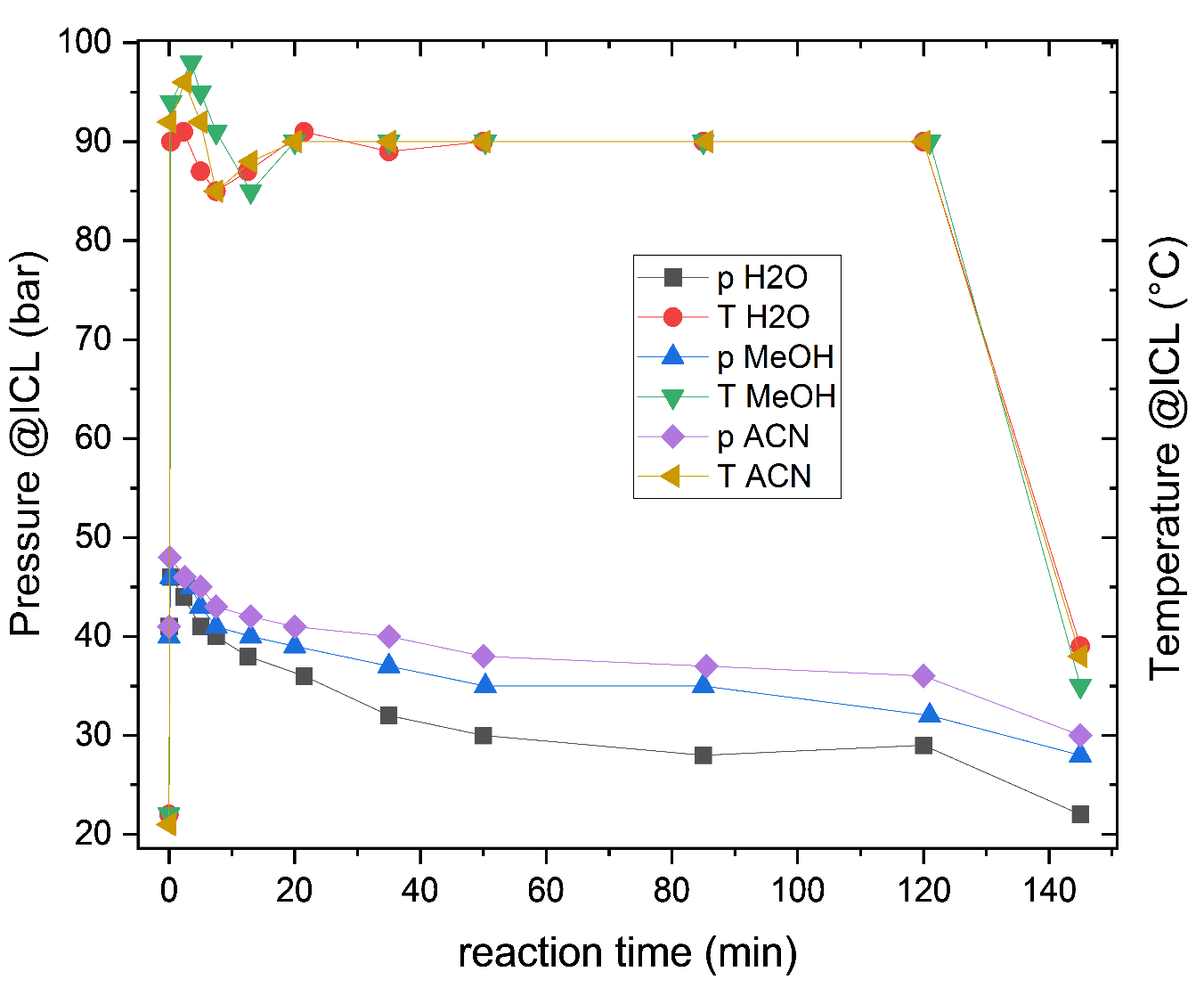


Figure S3: Pressure and Temperature-Time profile for reactions performed in Hallet Lab.

### Determination of observed reaction rates r_obs_


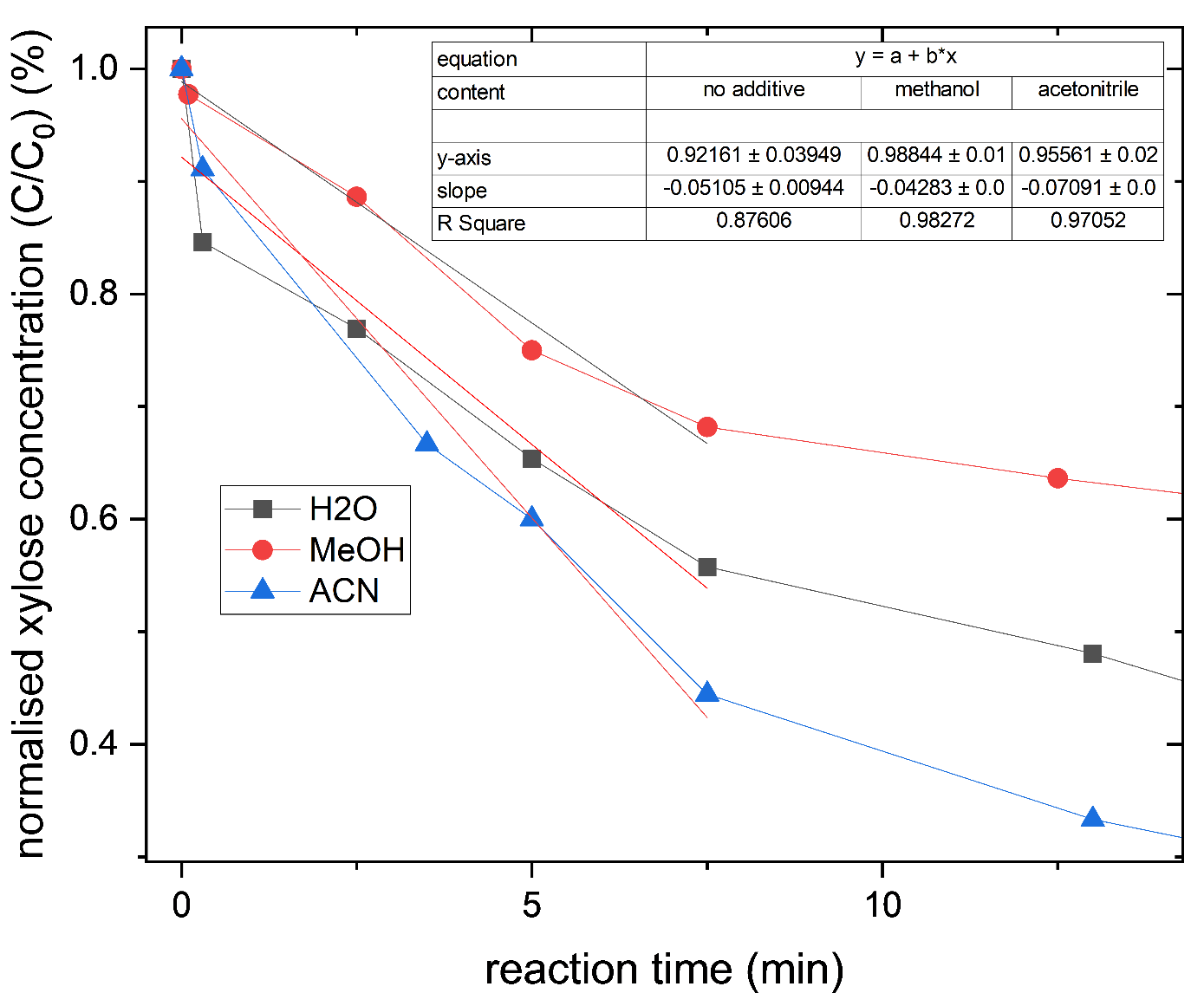


Figure S4: Normalized concentration-time profile for non-additive influenced xylose oxidation. Reaction conditions: Reaction at Hallet lab, c(H_5_PV_2_Mo_10_O_40_) = 35 mmol/L, c(Xylose) = 50 mmol/L, p_O2, start_ = 48 bar, V_stirrer_ = 1500 rpm (standard Parr impeller), solvent composition: 90:10 vol.% H_2_O:MeCN, 90:10 vol.% H_2_O:MeOH, 100 % H_2_O; T = 90 °C, t = 120 min, V = 30 mL.


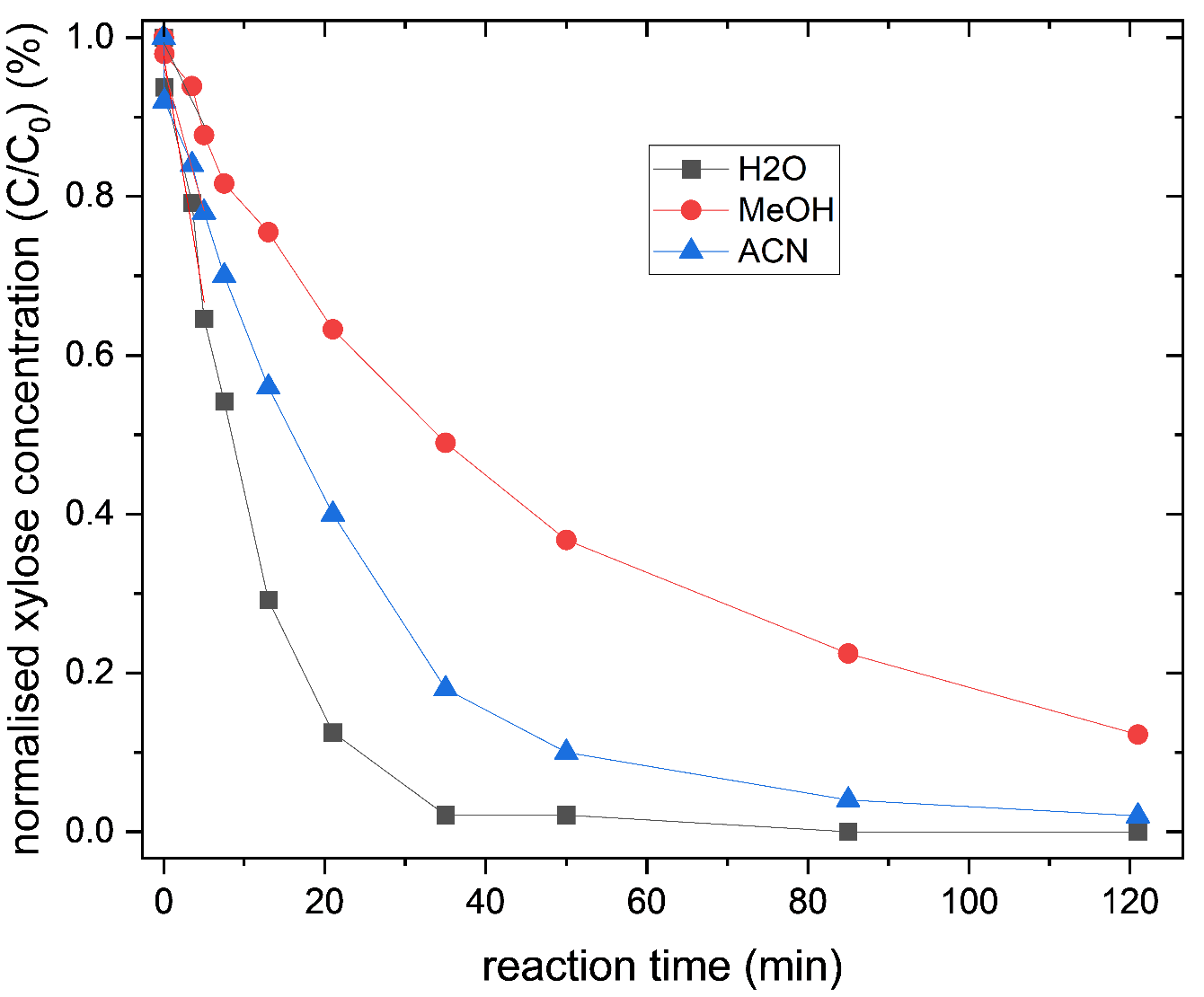


Figure S5: normalised xylose concentration again reaction time for investigated solvent systems. Reaction conditions: Set up at Albert Lab, c(H_5_PV_2_Mo_10_O_40_) = 35 mmol/L, c(Xylose) = 50 mmol/L, p_O2, start_ = 48 bar, V_stirrer_ = 1500 rpm (standard Parr impeller), solvent composition: 90:10 vol.% H_2_O:MeCN, 90:10 vol.% H_2_O:MeOH, 100 % H_2_O; T = 90 °C, t = 120 min, V = 45 mL.

### Exemplary HPLC Chromatogram


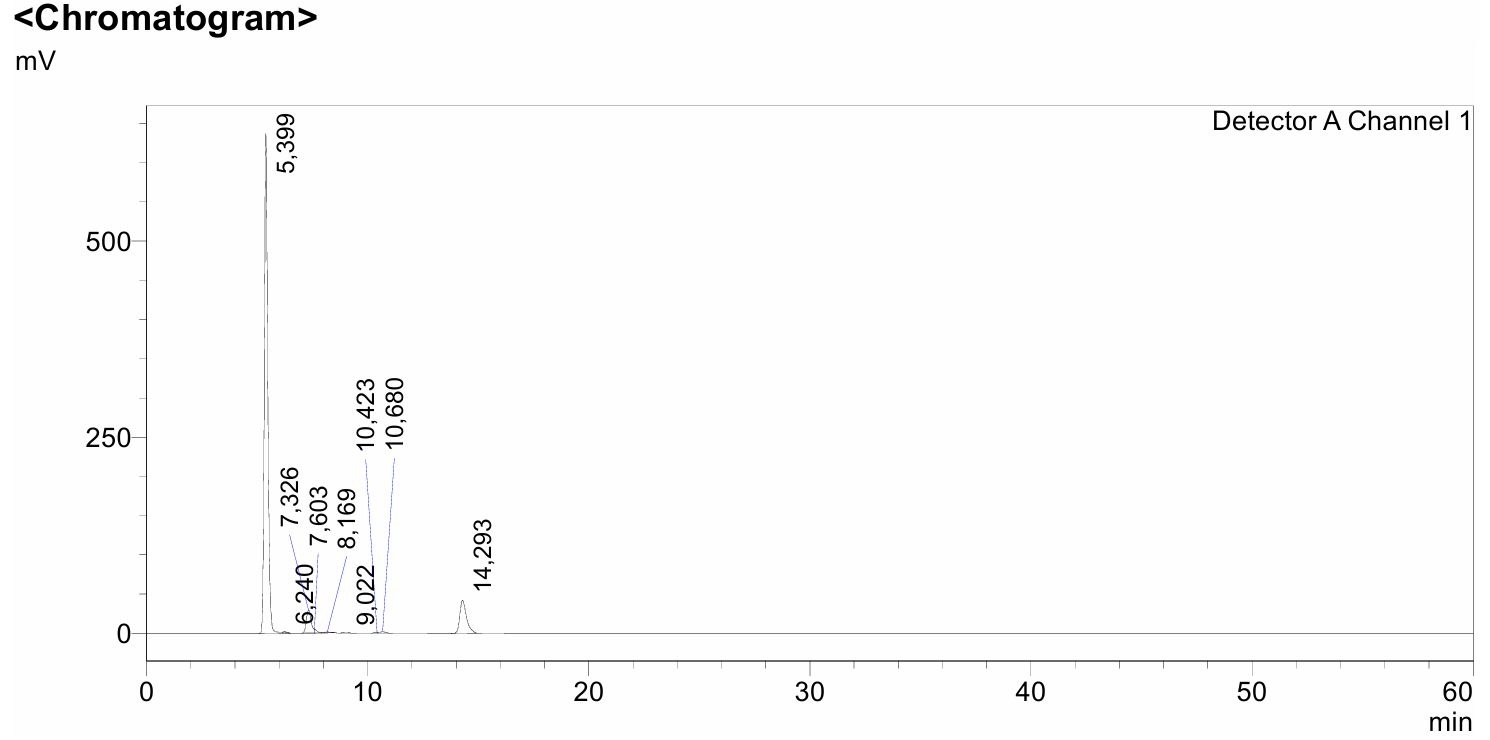


Figure S6: Exemplary HPLC chromatogram of methanolic influenced experiment. Ret.time (analyte): 5.4 min(injection/catalyst), 6.2 min (phosphate/catalyst), 7.3 min (xylose), 7.6 min (glyoxal), 8.2 min (glyceraldehyde), 9.0 min (glycolaldehyde), 10.4 min (formaldehyde), 10.7 min (formic acid), 14.3 min (methanol).

## 2.2 Full results for reactions in 3-fold screening plant (Albert lab at UHH)

### Catalytic results: pure aqueous experiment

A CO_2_ yield of 43.1 % was reached.

Table S4: Conversion and yield for non-additive experiment. Reaction conditions: c(H_5_PV_2_Mo_10_O_40_) = 35 mmol/L, c(Xylose) = 50 mmol/L, p_O2, start_ = 50 bar, V_stirrer_ = 1500 rpm (standard Parr impeller), solvent composition: 100 % H_2_O; T = 90 °C, t = 120 min, V = 45 mL.

| **Reaction time (min)** | **X_xylose_ (%)** | **Y_Formic acid_ (%)** | **Y_Glyceraldehyde_ (%)** | **Y_Glycolaldehyde_ (%)** | **Y_Glyoxal_ (%)** | **Y_Formaldehyde_ (%)** | **Y_Glycolic acid_ (%)** |
| --- | --- | --- | --- | --- | --- | --- | --- |
| **0** | 0.00 | 0.00 | 0.00 | 0.00 | 0.00 | 0.00 | 0.00 |
| **0.1** | 6.25 | 0.00 | 0.00 | 0.00 | 0.00 | 0.00 | 0.00 |
| **3.5** | 20.83 | 4.32 | 4.86 | 0.00 | 0.00 | 0.00 | 0.00 |
| **5** | 35.42 | 9.19 | 6.49 | 1.08 | 0.00 | 0.00 | 0.00 |
| **7.5** | 45.83 | 12.97 | 8.11 | 1.08 | 0.00 | 0.00 | 2.16 |
| **13** | 70.83 | 25.41 | 8.11 | 2.16 | 0.00 | 0.00 | 5.41 |
| **20** | 87.50 | 37.30 | 4.86 | 2.16 | 0.00 | 0.00 | 6.49 |
| **35** | 97.92 | 46.49 | 1.62 | 1.08 | 0.00 | 0.00 | 6.49 |
| **50** | 97.92 | 48.11 | 0.00 | 1.08 | 0.00 | 0.00 | 4.32 |
| **85** | 100.00 | 48.43 | 0.00 | 0.00 | 0.00 | 0.00 | 3.37 |
| **121** | 100.00 | 48.65 | 0.00 | 0.00 | 0.00 | 0.00 | 2.16 |

### Catalytic results: MeOH influenced reaction

Methyl formate is cleaved into methanol and formic acid during acidic HPLC measurement. Methanol is partly converted during the reaction mainly to formaldehyde. A CO_2_ yield of 1.1 % was achieved.

Table S5: Conversion and yield for methanolic influenced experiment. Reaction conditions: c(H_5_PV_2_Mo_10_O_40_) = 35 mmol/L, c(Xylose) = 50 mmol/L, p_O2, start_ = 50 bar, V_stirrer_ = 1500 rpm (standard Parr impeller), solvent composition: 90:10 % H_2_O:MeOH; T = 90 °C, t = 120 min, V = 45 mL.

| **Reaction time (min)** | **X_xylose_ (%)** | **Y_Formic acid_ (%)** | **Y_Glyceraldehyde_ (%)** | **Y_Glycolaldehyde_ (%)** | **Y_Glyoxal_ (%)** | **Y_Formaldehyde_ (%)** | **Y_Glycolic acid_ (%)** | **X_Methanol_ (%)** |
| --- | --- | --- | --- | --- | --- | --- | --- | --- |
| **0** | 0.00 | 0.00 | 0.00 | 0.00 | 0.00 | 0.00 | 0.00 | 0.00 |
| **0.1** | 2.04 | 0.00 | 1.22 | 0.00 | 0.00 | 0.00 | 0.00 | 1.81 |
| **3.5** | 6.12 | 2.04 | 1.22 | 0.00 | 0.00 | 1.22 | 0.00 | 2.71 |
| **5** | 12.24 | 3.27 | 2.45 | 0.82 | 3.12 | 1.22 | 0.00 | 3.47 |
| **7.5** | 18.37 | 4.49 | 2.45 | 0.82 | 5.46 | 1.63 | 0.82 | 4.37 |
| **13** | 24.49 | 8.16 | 2.45 | 2.45 | 5.46 | 1.63 | 2.45 | 5.58 |
| **20** | 36.73 | 12.24 | 3.67 | 6.53 | 10.15 | 2.86 | 3.27 | 6.68 |
| **35** | 51.02 | 24.08 | 3.67 | 8.98 | 9.36 | 4.90 | 4.08 | 8.14 |
| **50** | 63.27 | 36.73 | 3.67 | 10.61 | 10.15 | 6.12 | 4.90 | 9.30 |
| **85** | 77.55 | 55.92 | 2.45 | 10.61 | 8.58 | 8.98 | 4.08 | 12.01 |
| **121** | 87.76 | 71.43 | 2.45 | 8.16 | 6.24 | 10.61 | 3.27 | 13.92 |

### Catalytic results: MeCN influenced reaction

Acetonitrile is partly converted during the reaction. A CO_2_ yield of 8.6 % was achieved.

Table S6: Conversion and yield for acetonitrile influenced experiment. Reaction conditions: c(H_5_PV_2_Mo_10_O_40_) = 35 mmol/L, c(Xylose) = 50 mmol/L, p_O2, start_ = 50 bar, V_stirrer_ = 1500 rpm (standard Parr impeller), solvent composition: 90:10 % H_2_O:MeCN; T = 90 °C, t = 120 min, V = 45 mL.

| **Reaction time (min)** | **X_xylose_ (%)** | **Y_Formic acid_ (%)** | **Y_Glyceraldehyde_ (%)** | **Y_Glycolaldehyde_ (%)** | **Y_Glyoxal_ (%)** | **Y_Formaldehyde_ (%)** | **Y_Glycolic acid_ (%)** | **X_Acetonitrile_ (%)** |
| --- | --- | --- | --- | --- | --- | --- | --- | --- |
| **0** | 0.00 | 0.00 | 0.00 | 0.00 | 0.00 | 0.00 | 0.00 | 0.00 |
| **0.1** | 8.00 | 1.20 | 1.20 | 0.00 | 0.00 | 0.00 | 0.00 | 2.28 |
| **3.5** | 16.00 | 3.60 | 3.60 | 0.00 | 0.00 | 0.00 | 0.00 | 4.21 |
| **5** | 22.00 | 6.00 | 3.60 | 0.80 | 0.00 | 0.00 | 0.00 | 5.51 |
| **7.5** | 30.00 | 9.20 | 4.80 | 1.60 | 0.00 | 0.00 | 0.80 | 6.76 |
| **13** | 44.00 | 16.40 | 4.80 | 2.40 | 0.00 | 0.00 | 2.40 | 7.21 |
| **20** | 60.00 | 25.60 | 4.80 | 3.20 | 0.00 | 0.40 | 3.20 | 7.84 |
| **35** | 82.00 | 40.00 | 3.60 | 3.20 | 0.00 | 1.20 | 4.00 | 8.06 |
| **50** | 90.00 | 48.80 | 1.20 | 2.40 | 4.02 | 1.60 | 4.80 | 8.15 |
| **85** | 96.00 | 57.60 | 0.00 | 1.60 | 2.41 | 1.60 | 4.00 | 9.58 |
| **121** | 98.00 | 61.60 | 0.00 | 0.80 | 0.80 | 1.60 | 3.20 | 12.90 |

# **3. Additional computational results**

## 3.1 Binding positions to the POM

Figure S7. Binding positions in the HPA-2 catalyst

## 3.2 Binding energies and orientations

Table S7. Calculated binding energies of oxidised and reduced HPA-2 with MeCN and MeOH, calculated at the r^2^SCAN-3c level.

| **Position** | **MeCN (ox)** | **MeCN (red)** | **MeOH (ox)** | **MeOH (red)** |
| --- | --- | --- | --- | --- |
| 1 | -23.8 | -33.3 | -31.6 | -42.0 |
| 2 | -19.4 | -28.6 | -27.5 | -36.1 |
| 3 | -22.8 | -26.1 | -33.9 | -38.0 |
| 4 | -20.1 | -24.3 | -27.9 | -29.8 |
| 5 | -22.2 | -26.8 | -33.0 | -32.3 |
| 6 | -21.5 | -29.3 | -26.7 | -36.6 |
| 7 | -20.6 | -28.3 | -31.4 | -33.1 |
| 8 | -18.5 | -28.3 | -31.9 | -34.6 |
| 9 | -20.7 | -27.7 | -25.4 | -24.8 |
| 10 | -20.0 | -26.0 | -25.3 | -25.5 |
| 11 | -10.8 | -21.1 | -26.2 | -28.8 |
| 12 | -19.4 | -26.2 | -30.3 | -36.8 |
| 13 | -21.5 | -28.0 | -23.4 | -29.7 |
| 14 | -20.6 | -28.5 | -32.8 | -36.4 |
| 15 | -20.2 | -27.8 | -32.1 | -37.1 |
| 16 | -21.0 | -27.0 | -33.9 | -39.6 |
| 17 | -18.4 | -26.2 | -32.2 | -33.8 |
| 18 | -22.8 | -28.5 | -27.9 | -26.8 |
| 19 | -23.4 | -28.5 | -36.2 | -33.2 |
| 20 | -17.4 | -28.8 | -17.9 | -28.0 |
| 21 | -21.1 | -26.6 | -31.0 | -35.4 |


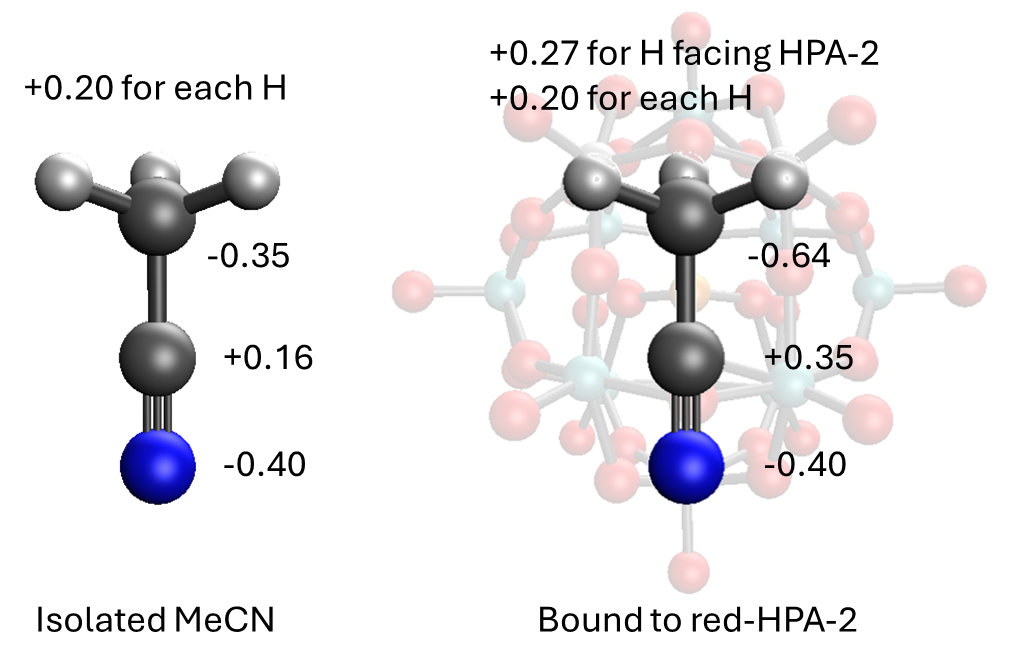


Figure S8. Calculated r^2^SCAN-3c Mulliken partial charges of isolated MeCN (left) and MeCN when bound to reduced HPA-2 (right). Partial charges are truncated to 2sf.

# **4. Spectroscopic and electrochemical investigation of HPA-2**

## 4.1 Preparation of catalyst solution and pH values for spectroscopic analysis

Three solutions corresponding to the performed reactions were prepared. Solvent composition is either 100 % DI-water or a mixture of 90 vol.% DI-water mixed with 10 vol.% MeOH orMeCN. Samples were prepared by adding 150 mg HPA-2 catalyst in 2 mL solvent (c_HPA‑2_ = 35 mmol/L) and the pH value was measured with a SenTix Mic micro pH probe. Table S1 shows corresponding pH values resulting of the different mixtures. Sample solutions with isotope labelling were performed with the same parameters.

Table S8: Resulting pH values for each prepared sample.

| **Sample** | **Resulting pH value (-)** |
| --- | --- |
| 100 % H_2_O | 0.965 |
| H*_2_*O:MeOH=9:1 | 0.992 |
| H*_2_*O:MeCN=9:1 | 0.960 |

## 4.2 NMR Spectroscopy

The catalyst was additionally characterized by ^51^V- and ^31^P-NMR spectroscopy using a Bruker *Avance III* 600 MHz spectrometer. Solution prepared as shown in beforehand part. ^31^P-Spectra and ^51^V-Spectra can be found in Figure S10.

| 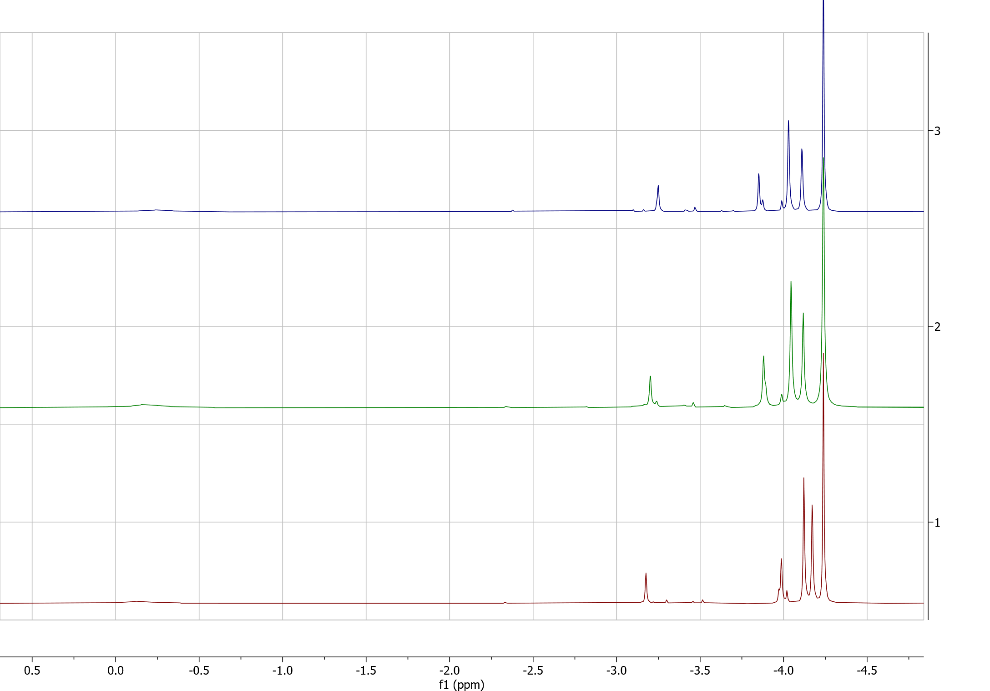  H_2_O  H_2_O:MeOH=9:1  H_2_O:MeCN=9:1 | 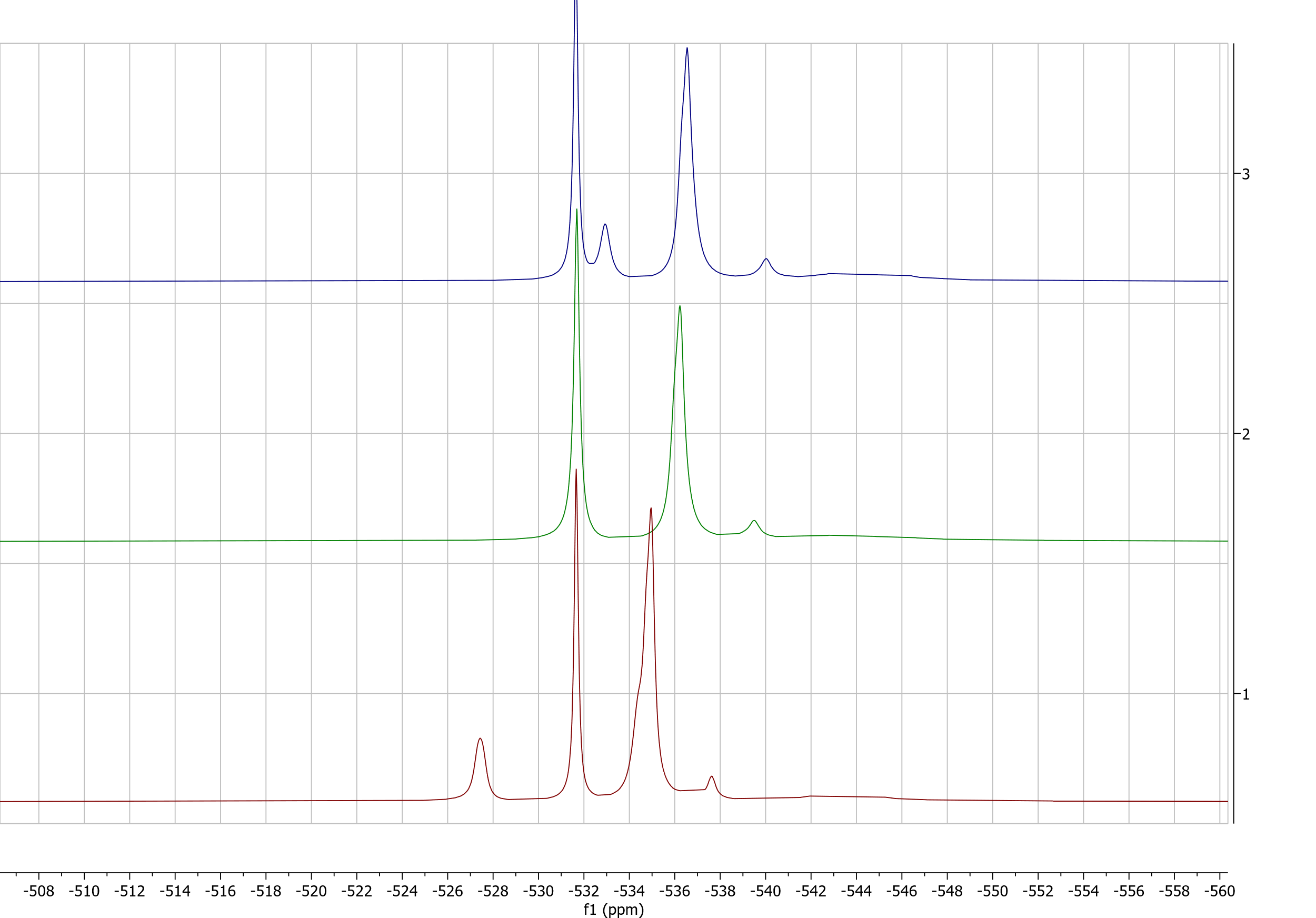 |
| --- | --- |

Figure S9: ^31^P (left) and ^51^V (right)-NMR spectra of HPA-2 in different solvent mixtures.

## 4.3 UV-Vis Spectroscopy

The catalyst in solution was additionally characterized UV-Vis spectroscopy using a Cary 60 Spectrophotometer by Agilent. Spectra can be found in Figure S9. UV-Vis spectra don’t correlate with pH-value differences and show therefore only the V^+IV^ content.


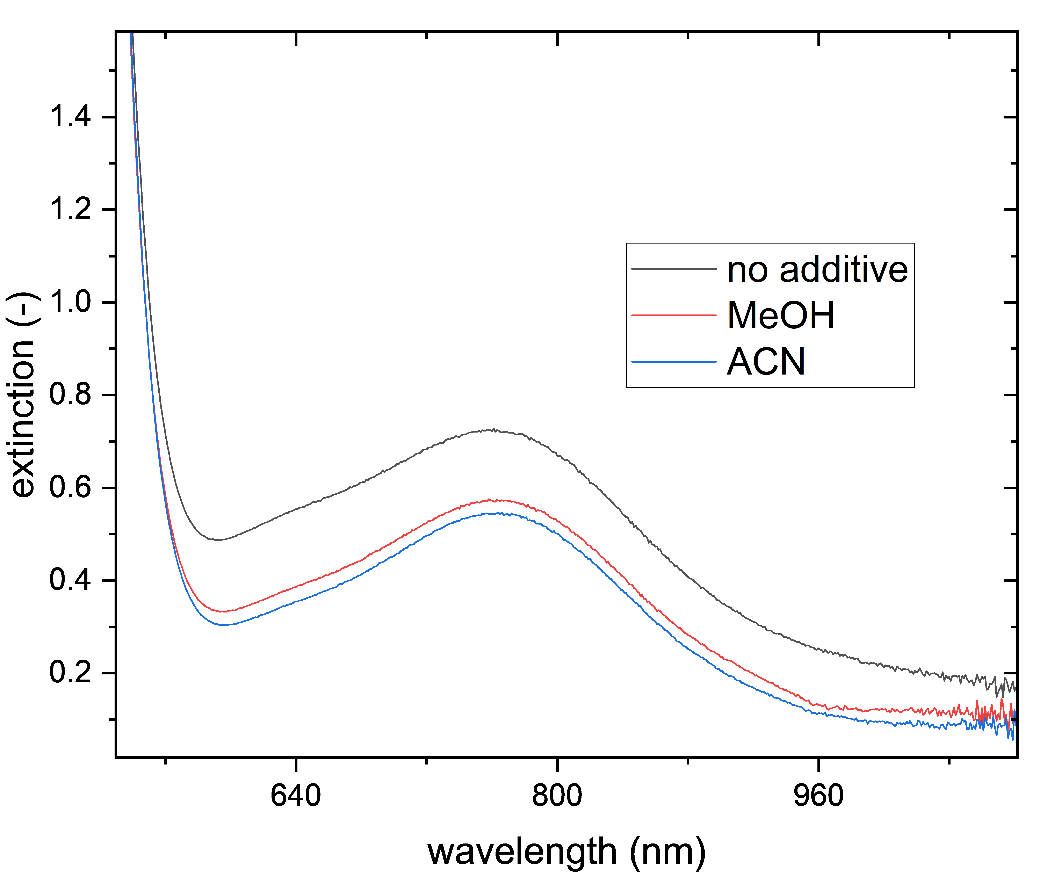


Figure S10: UV-Vis spectra of reference catalyst-solvent solutions.

## 4.4 FT-IR Spectroscopy (solid state)


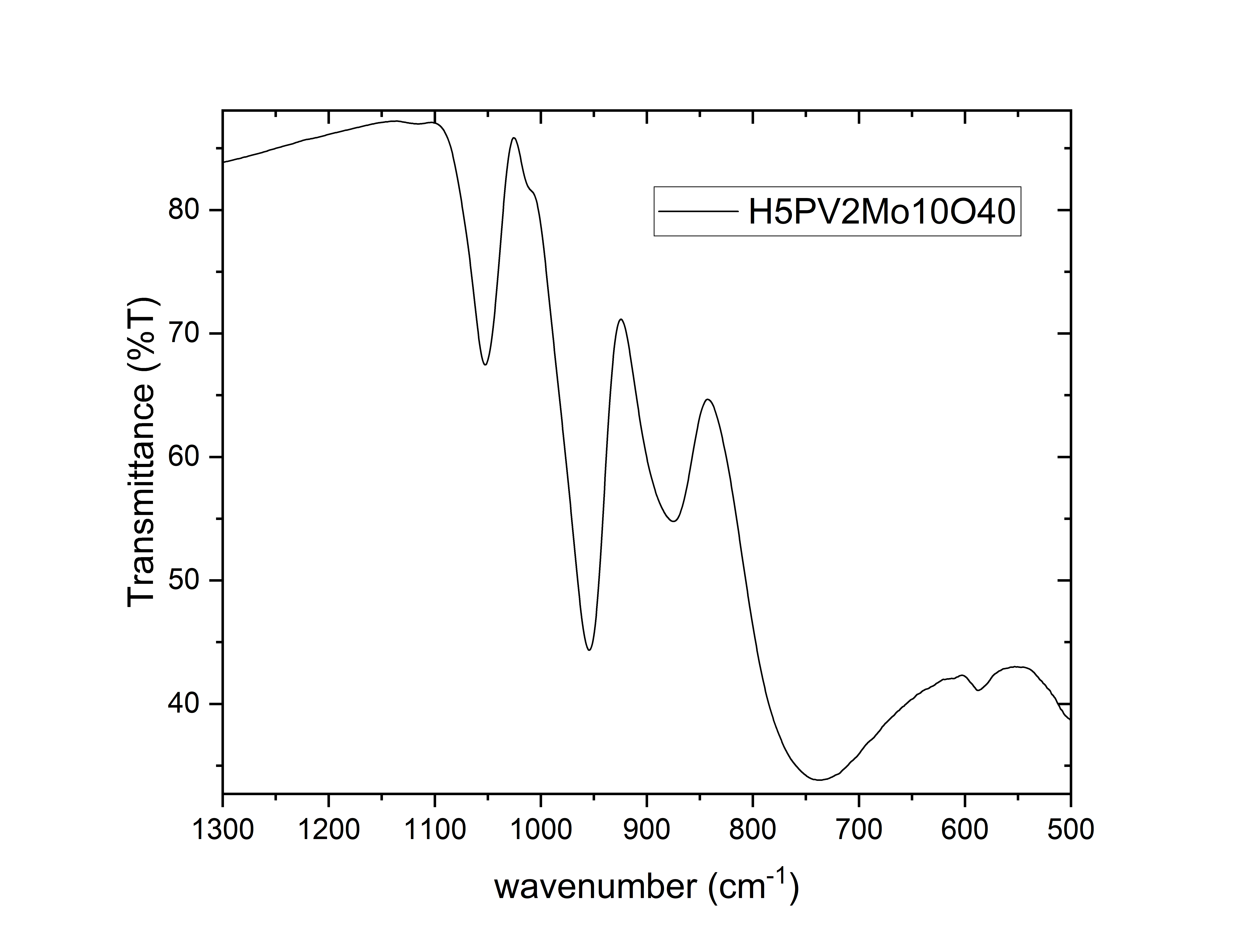


Figure S11: IR Spectrum of HPA-2 catalyst used in this study.

## 4.5 Electrochemical measurements

Figure S12: Cyclic voltammograms of HPA-2 (1 mmol/L) in H_2_O (0.5 M H_2_SO_4_ as supporting electrolyte) at pH 0.6; different scan rates (arrow indicates scanning direction).

 Figure S13: Comparison of cyclic voltammograms of HPA-2 (1 mmol/L) in H_2_O, using 0.5 M H_2_SO_4_ and 0.1 M HCl as supporting electrolytes; scan rate100 mV/s (arrows indicate scanning direction).

 *Figure S14: Cyclic voltammograms of HPA-2 (1 mmol/L) in H_2_O (0.1 M HCl as supporting electrolyte) at pH 1; different scan rates (arrow indicates scanning direction).*

 *Figure S15: Cyclic voltammograms of HPA-2 (1 mmol/L) in H_2_O:MeOH 9:1 (0.1 M HCl as supporting electrolyte) at pH 1; different scan rates (arrow indicates scanning direction).*

*Figure S16: Cyclic voltammograms of HPA-2 (1 mmol/L) in H_2_O:MeCN 9:1 (0.1 M HCl as supporting electrolyte) at pH 1; different scan rates (arrow indicates scanning direction).*

*Figure S17: Comparison of cyclic voltammograms of HPA-2 (1 mmol/L) in various solvent composition, using 0.1 M HCl as supporting electrolyte; scan rate100 mV/s (arrow indicates scanning direction).*

Figure S18: Comparison of cyclic voltammograms of HPA-2 (1 mmol/L) in various solvent composition, using 0.1 M HCl as supporting electrolyte; scan rate100 mV/s (arrow indicates scanning direction).

## 4.6 EPR Spectroscopy

*General considerations*

The room-temperature X-band CW-EPR spectra of the HPA-2 samples were recorded using a non-saturating microwave power of 20 mW, a field modulation of 0.25 mT at 100 kHz, a conversion time of 40.96 ms, a time constant of 10.24 ms and a magnetic field sweep rate of 1.2 mT/s. Details can be found in Figures S19-S21.

The frozen-matrix X-band CW-EPR spectra of the HPA-2 samples were recorded at a temperature of 100 K using a non-saturating microwave power of 2 mW, a field modulation of 0.25 mT at 100 kHz, a conversion time of 40.96 ms, a time constant of 10.24 ms and a magnetic field sweep rate of 1.5 mT/s.

The EPR spectra were processed using home-written scripts running on MATLAB R2024b and based on EasySpin v6.0.12.^4^ The room-temperature X-band CW, frozen-matrix Q-band field sweeps and Q-band pulse EPR spectra were simulated using the *garlic*, *pepper* and *saffron* functions, respectively. The details of the simulations are reported in each section.

X-band continuous-wave EPR spectroscopy

For each solvent system, the frozen-matrix X-band CW-EPR spectra recorded at 100 K were simulated by a global fitting approach by linking the *g* and *A* principal values throughout the sample series (see Tables S9-S11) and optimising the line broadening parameters for each individual spectrum. The results are displayed in Figure S19 and the fitting parameters are summarised in Table S9 and Table S10.


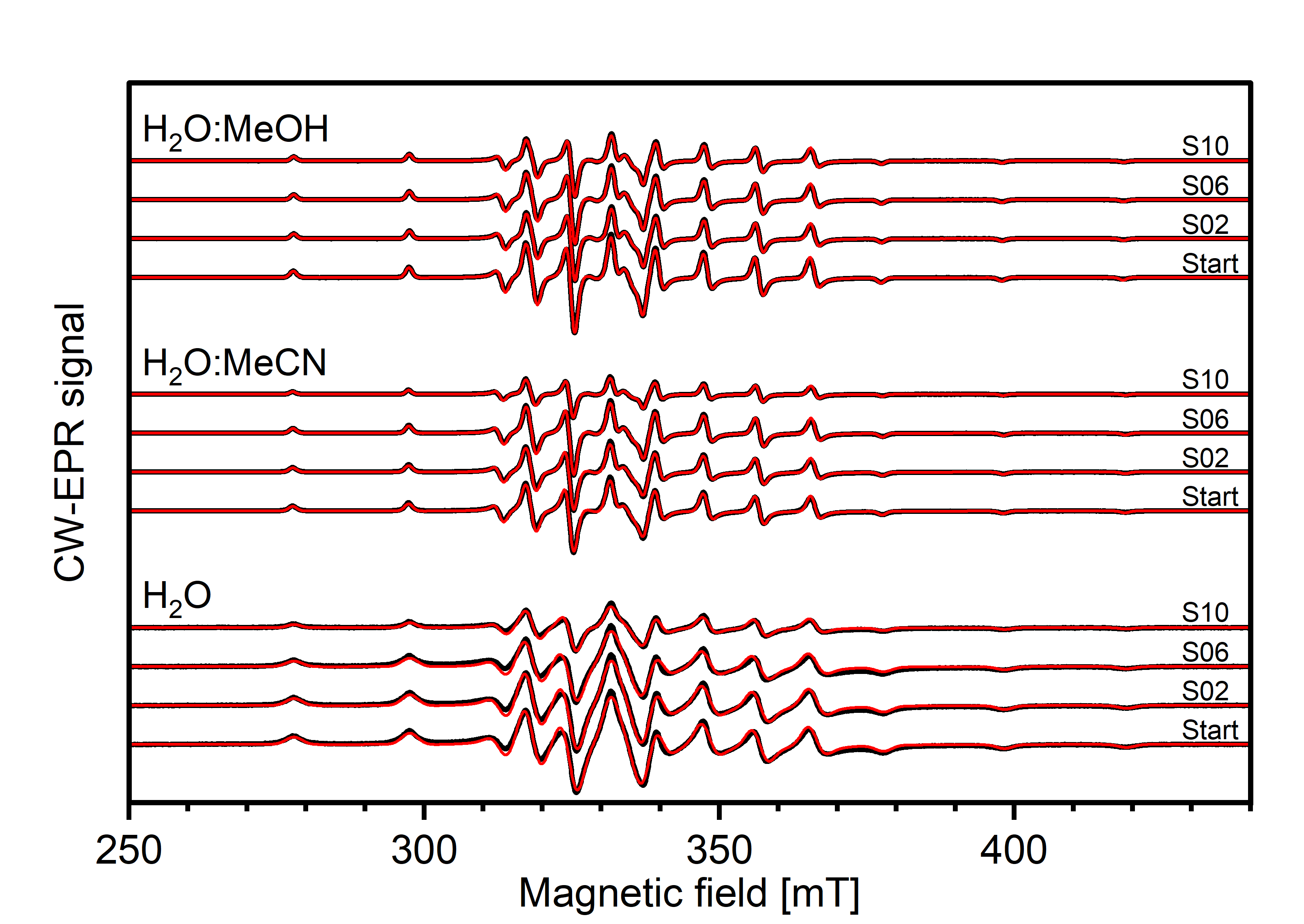


*Figure S19: Sample frozen-matrix X-band CW-EPR spectra (black traces) and simulations (red traces).*

For the simulation of the room-temperature X-band CW-EPR spectra, the values of *g*_1_ = (2*g*_∥_-*2*g_⟂_)/6 and *a*_1_ = (2*A*_∥_-*2*A_⟂_)/6, defining the axial part of the corresponding interactions, were kept fixed and equal to the values determined from the best fit of the frozen-matrix spectra. A preliminary fit, in which for a given solvent mixture the rotational correlation time and the linewidth parameters were allowed to change for each spectrum, showed a neglible variation of the rotational correlation time throughout the sample series. With this in mind, the best fits of the experimental spectra, displayed in Figure S20 with the relevant parameters summarised in Table S10 and Table S11, were obtained by setting the latter as a global parameter.


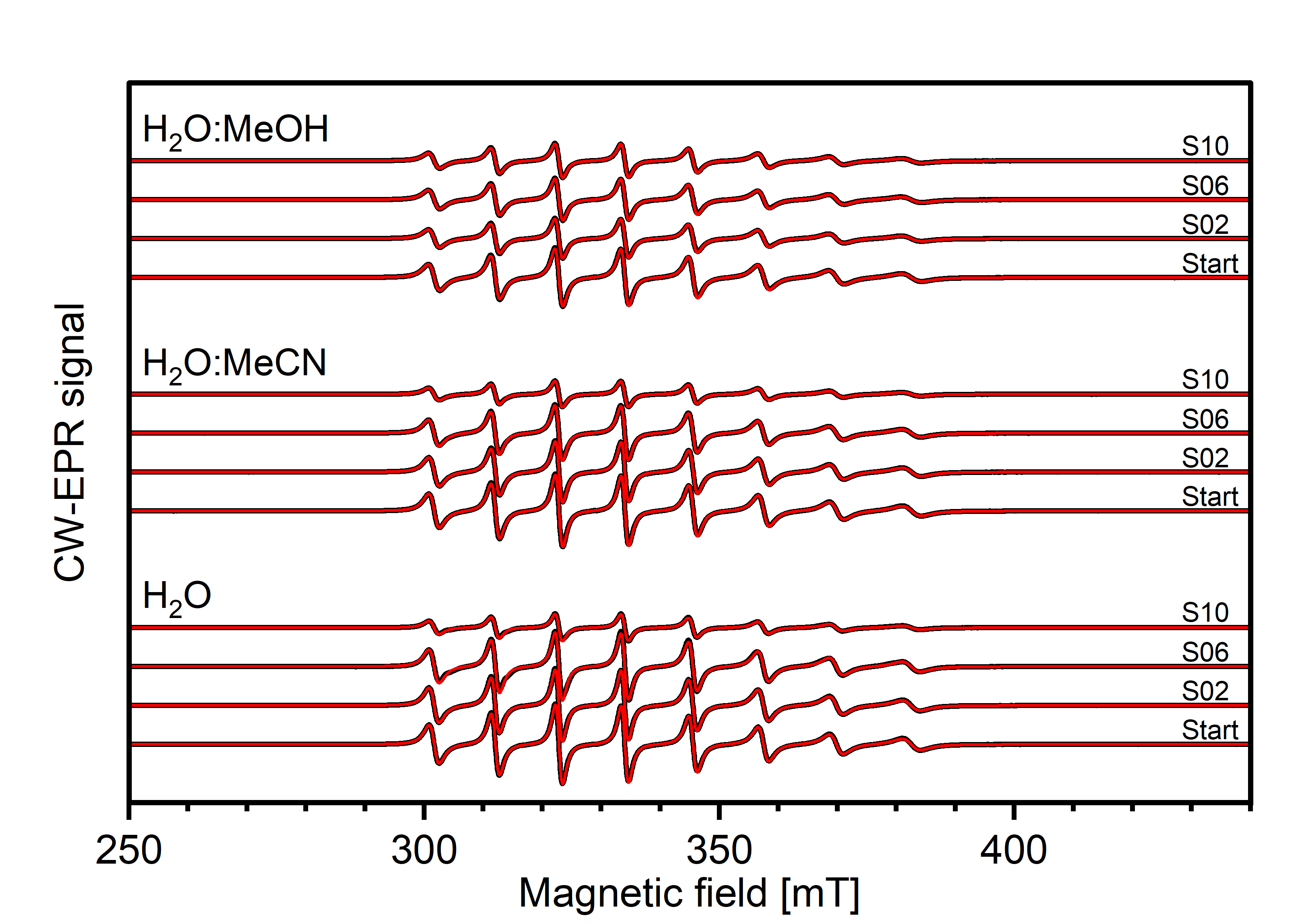


*Figure S20: Sample room-temperature X-band CW-EPR spectra (black traces) and simulations (red traces).*

*Table S9: Global fitting parameters obtained from the simulation of the frozen-matrix and room-temperature CW-EPR spectra for each solvent system.*

|  | Frozen matrix | | | | Room temperature | | |
| --- | --- | --- | --- | --- | --- | --- | --- |
| Solvent system | *g*_⟂_ | *g*_∥_ | *A*_⟂_ | *A*_∥_ | *g*_iso_ | *a*_iso_ | *τ*_c_ |
| H_2_O | 1.9781 | 1.9328 | 206 MHz | 545 MHz | 1.9653 | 318 MHz | 57 ps |
| H_2_O:MeCN | 1.9782 | 1.9334 | 208 MHz | 545 MHz | 1.9653 | 318 MHz | 61 ps |
| H_2_O:MeOH | 1.9779 | 1.9343 | 206 MHz | 543 MHz | 1.9653 | 318 MHz | 67 ps |

*Table S10: Sample-dependent fitting parameters of the frozen-matrix spectra. The linewidth for a given orientation* $(n_{x},n_{y},n_{z})=(\sin(\theta)\cos(\varphi),\sin(\theta)\sin(\varphi),\cos(\theta))$ *of the magnetic field vector in the principal frame of the* ***g*** *and* ***A*** *tensors is obtained as* $LW(\theta)=\sqrt{{LW}_{\perp}^{2}{sin}^{2}(\theta)+{LW}_{\parallel}^{2}{cos}^{2}(\theta)}$*.*

|  | H_2_O | | H_2_O:MeCN | | H_2_O:MeOH | |
| --- | --- | --- | --- | --- | --- | --- |
| Sample | LW_⊥_ | LW_∥_ | LW_⊥_ | LW_∥_ | LW_⊥_ | LW_∥_ |
| Start | 3.31 mT | 3.20 mT | 1.75 mT | 1.65 mT | 1.59 mT | 1.35 mT |
| S01 | 3.20 mT | 3.10 mT | 1.69 mT | 1.57 mT | 1.48 mT | 1.21 mT |
| S02 | 3.28 mT | 3.16 mT | 1.69 mT | 1.57 mT | 1.49 mT | 1.22 mT |
| S03 | 3.37 mT | 3.25 mT | 1.69 mT | 1.57 mT | 1.49 mT | 1.23 mT |
| S04 | 3.44 mT | 3.30 mT | 1.69 mT | 1.57 mT | 1.51 mT | 1.25 mT |
| S05 | 3.43 mT | 3.32 mT | 1.66 mT | 1.53 mT | 1.51 mT | 1.25 mT |
| S06 | 3.34 mT | 3.24 mT | 1.59 mT | 1.42 mT | 1.51 mT | 1.24 mT |
| S07 | 3.01 mT | 2.96 mT | 1.54 mT | 1.34 mT | 1.50 mT | 1.24 mT |
| S08 | 2.80 mT | 2.75 mT | 1.49 mT | 1.27 mT | 1.48 mT | 1.21 mT |
| S09 | 2.62 mT | 2.56 mT | 1.44 mT | 1.18 mT | 1.47 mT | 1.20 mT |
| S10 | 2.62 mT | 2.57 mT | 1.43 mT | 1.17 mT | 1.45 mT | 1.18 mT |

*Table S11: Sample-dependent fitting parameters of the room-temperature spectra. The convolution of a Gaussian (G) and a Lorentzian (L) function (Voigtian broadening model) has been used to describe the EPR line shape in the limit of τ_c_ → 0.*

|  | H_2_O | | H_2_O:MeCN | | H_2_O:MeOH | |
| --- | --- | --- | --- | --- | --- | --- |
| Sample | LW_G_ | LW_L_ | LW_G_ | LW_L_ | LW_G_ | LW_L_ |
| Start | 0.48 mT | 0.12 mT | 0.47 mT | 0.12 mT | 0.46 mT | 0.12 mT |
| S01 | 0.47 mT | 0.12 mT | 0.47 mT | 0.13 mT | 0.46 mT | 0.12 mT |
| S02 | 0.46 mT | 0.13 mT | 0.46 mT | 0.13 mT | 0.46 mT | 0.09 mT |
| S03 | 0.45 mT | 0.16 mT | 0.46 mT | 0.13 mT | 0.46 mT | 0.08 mT |
| S04 | 0.43 mT | 0.20 mT | 0.44 mT | 0.15 mT | 0.46 mT | 0.09 mT |
| S05 | 0.39 mT | 0.24 mT | 0.44 mT | 0.15 mT | 0.47 mT | 0.09 mT |
| S06 | 0.37 mT | 0.25 mT | 0.44 mT | 0.15 mT | 0.46 mT | 0.12 mT |
| S07 | 0.36 mT | 0.25 mT | 0.43 mT | 0.16 mT | 0.48 mT | 0.09 mT |
| S08 | 0.37 mT | 0.24 mT | 0.45 mT | 0.13 mT | 0.48 mT | 0.07 mT |
| S09 | 0.39 mT | 0.20 mT | 0.45 mT | 0.14 mT | 0.47 mT | 0.07 mT |
| S10 | 0.39 mT | 0.20 mT | 0.45 mT | 0.14 mT | 0.48 mT | 0.06 mT |

Quantitative information was extracted from the room-temperature spectra by calibrating the response against a series of copper(II) tetrakis-imidazole dilutions (see Figure S21). These latter samples were measured using a non-saturating microwave power of 20 mW, a field modulation of 0.25 mT at 100 kHz, a conversion time of 40.96 ms, a time constant of 10.24 ms and a magnetic field sweep rate of 1.2 mT/s.

| 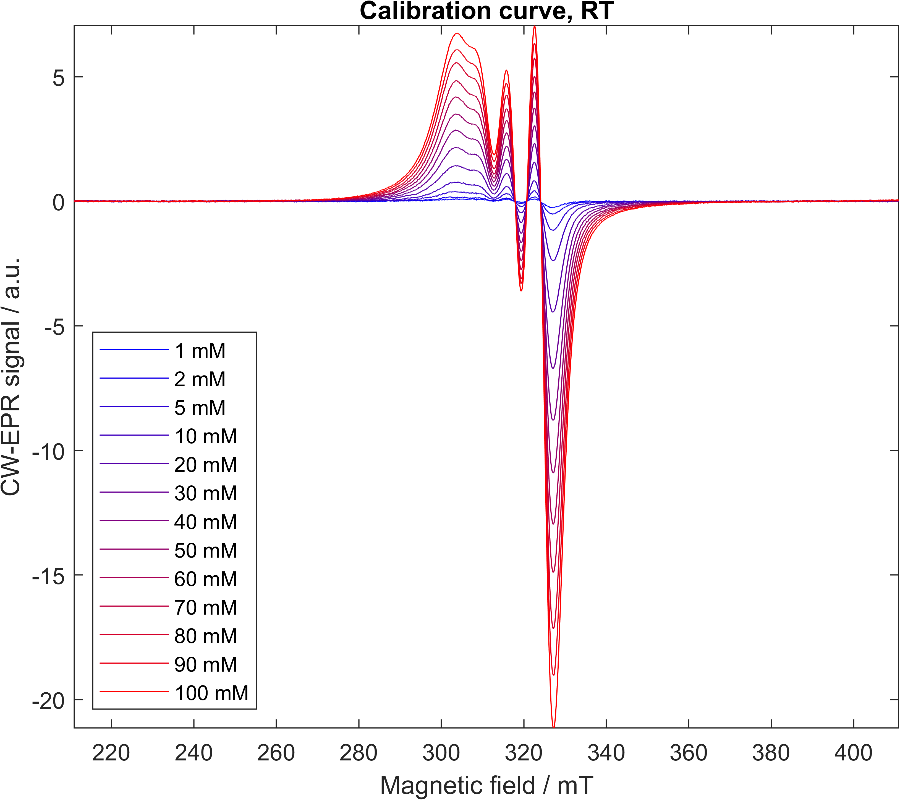 | 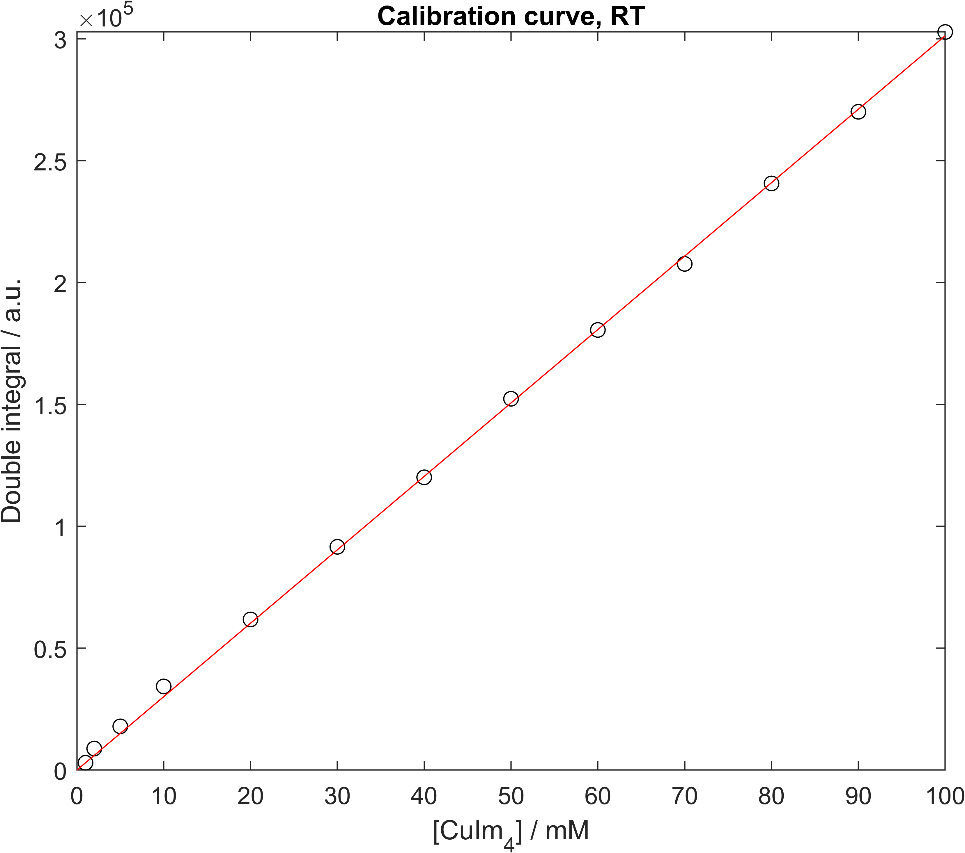 |
| --- | --- |

*Figure S21: Calibration of the response of the spectrometer at room temperature response against a series of copper(II) tetrakis-imidazole dilutions.*

Q-band pulse EPR spectroscopy

All the experiments were performed at 20 K using a shot repetition rate of 0.49 kHz (2.04 ms shot repetition time). Before each measurement, after loading the sample the resonator profile of the overcoupled resonator (Q ≈ 140, corresponding to a resonator bandwidth of ≈ 230 MHz FWHM) was measured and the frequency of the transmitter was placed in the centre of the resonator mode. Due to the very high sample concentration, the low-noise amplifier of the microwave bridge was bypassed. For all the experiments the stripline pulse-forming units (SPFUs) of the spectrometer were used to generate the microwave pulses.

Echo-detected field-swept EPR (EDFS-EPR) spectra were measured using the pulse sequence (π/2) – τ – (π) – τ – echo with the length of the π/2 and π pulses set to 16 ns and 32 ns, respectively, and an inter-pulse delay τ of 200 ns. A 2-step phase cycle (x)x was applied to the π/2 pulse^5^ and the echo was integrated using a gate of 100 ns centred at the echo maximum.

For the HYSCORE experiments the magnetic field was placed at the maximum of the absorption spectrum; this corresponds to selecting molecules whose x_M_/y_M_ axes are parallel to the applied static magnetic field (see Figure S22), whereby the {x_M_, y_M_, z_M_} molecular frame corresponds to the eigenframe of the ***g*** and ***A*** interactions.

| 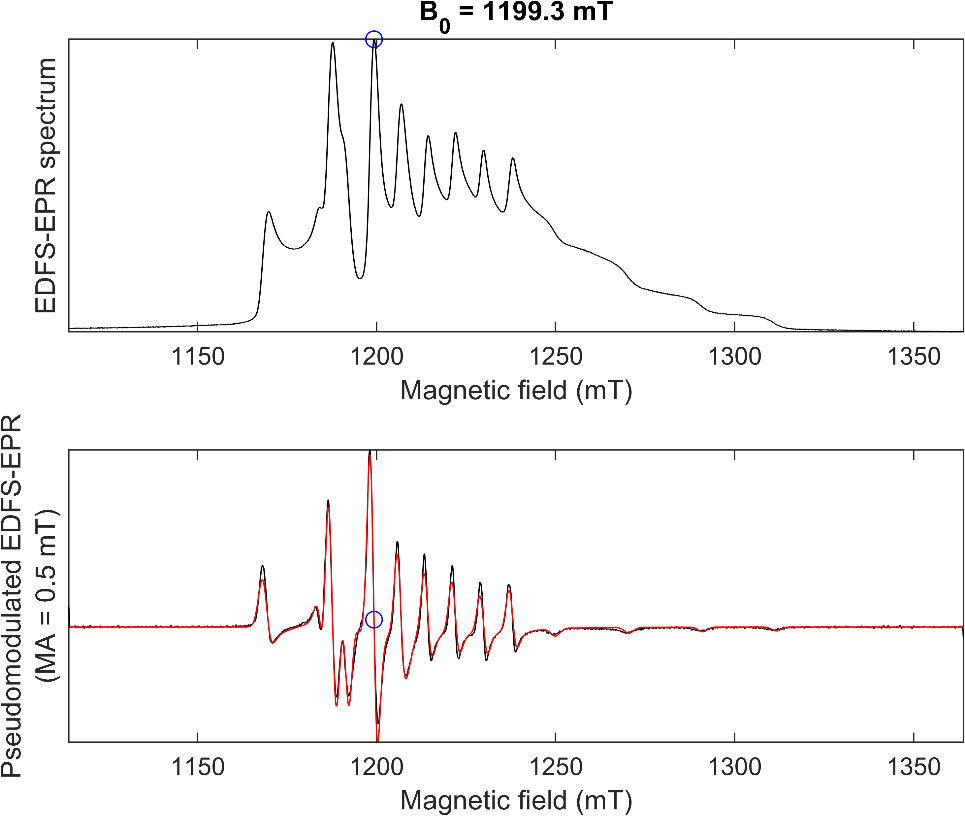 | 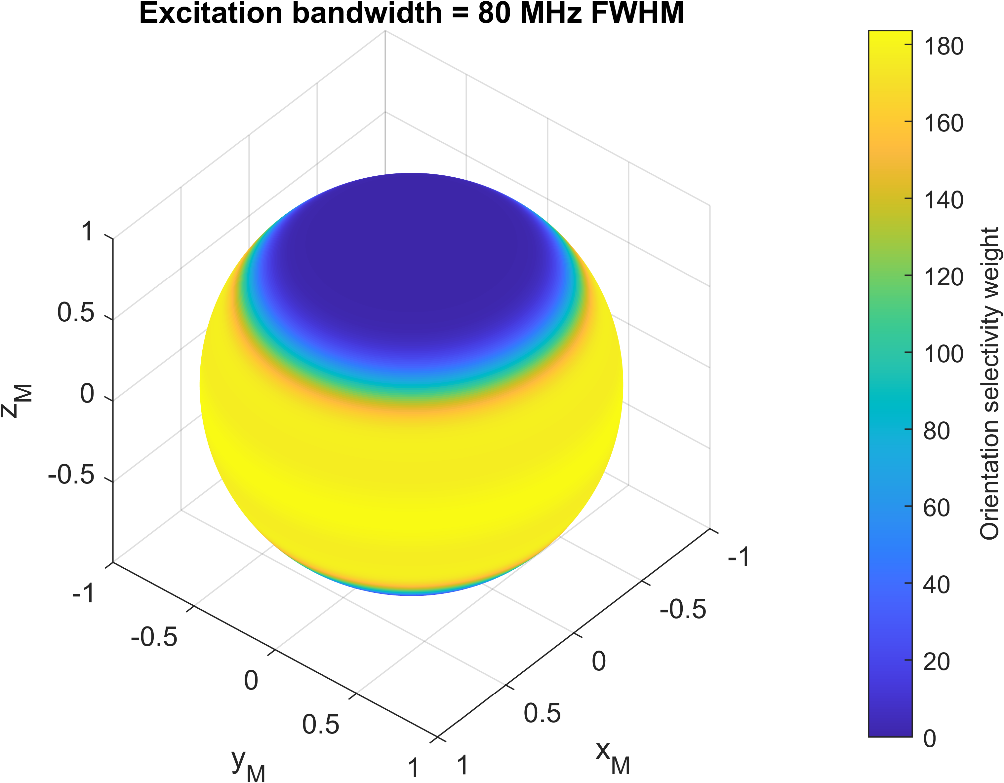 |
| --- | --- |

*Figure S22: Choice of the field position for the pulse EPR experiments. Left: EDFS-EPR spectrum (top) and the corresponding pseudomodulated spectrum (bottom, black) with the simulation (bottom, red; parameters: g_xy_ = 1.9792, g_z_ = 1.9336; A_xy_ = 208 MHz, A_z_ = 554 MHz; Γ_xy_ = 66 MHz FWHM, Γ_z_ = 79 MHz FWHM whereby Γ is the anisotropic linewidth introduced via Sys.HStrain). The blue circle highlights the field position at which the pulse EPR experiments have been performed. Right: orientation selectivity weights for a pulse with an excitation bandwidth of 80 MHz FWHM, corresponding to a 12 ns rectangular pulse, in the coordinate frame defined by the principal axes of the* ***g*** *and* ***A*** *interaction tensors.*

4-pulse HYSCORE^6^ experiments were performed using the pulse sequence

(π/2) – τ – (π/2) – *t*_1_ – (π) – *t*_2_ – (π/2) – τ – echo

in which the time intervals *t*_1_ and *t*_2_ were varied independently starting from 80 ns in steps of 8 ns each; 384 points were collected along each time dimension, corresponding to maximum *t*_1_ and *t*_2_ values of 3.144 µs. Unless stated otherwise, *τ* was set to 156 ns, corresponding to the 8^th^-order blind spot of the diagonal peak at the ^1^H nuclear Larmor frequency. The length of the π/2 and π pulses was set to 6 ns and 12 ns, respectively; the echo was integrated over a 10 ns gate. A 16-step phase cycle x[x](x)(x) was used to select the coherence transfer pathways yielding the desired signal.^5^ A typical acquisition took approximately 8 hours per spectrum.

The very short increment of the *t*_1_ and *t*_2_ time intervals, corresponding to a Nyquist frequency of ± 62.5 MHz on both the ν_1_ and ν_2_ axes, is required as ^1^H couplings (ν_L_(^1^H) ≈ 51 MHz at the chosen magnetic field) can be clearly detected and would otherwise result in foldback artefacts, complicating the analysis of the spectra.

The experimental time-domain traces were baseline-corrected with a 5^th^-order polynomial function along both time dimension, apodised with a Hamming window function, zero-filled to 2048 points and 2D Fourier transformed. The spectra are displayed as the absolute value of the 2D-FFT after symmetrisation about the diagonal ($S_{1}={(S*S^{\tau})}^{1/2}$ where *S* is the matrix obtained from the absolute value of the 2D-FFT of the HYSCORE data and *τ* denotes the matrix transpose).^7^

The full spectra, including the ^1^H region, are displayed in Figure S23. Due do the lack of peaks above 20 MHz, only the spectral region up to 25 MHz is shown.

| 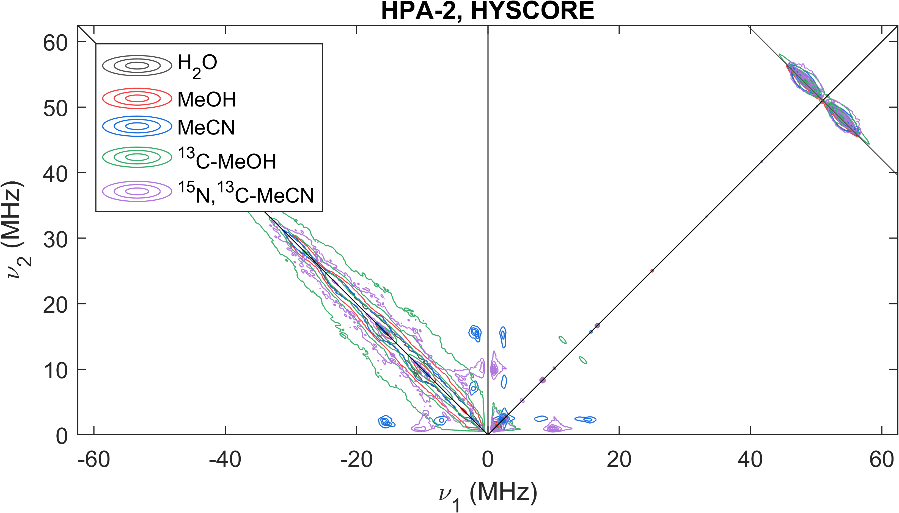 | 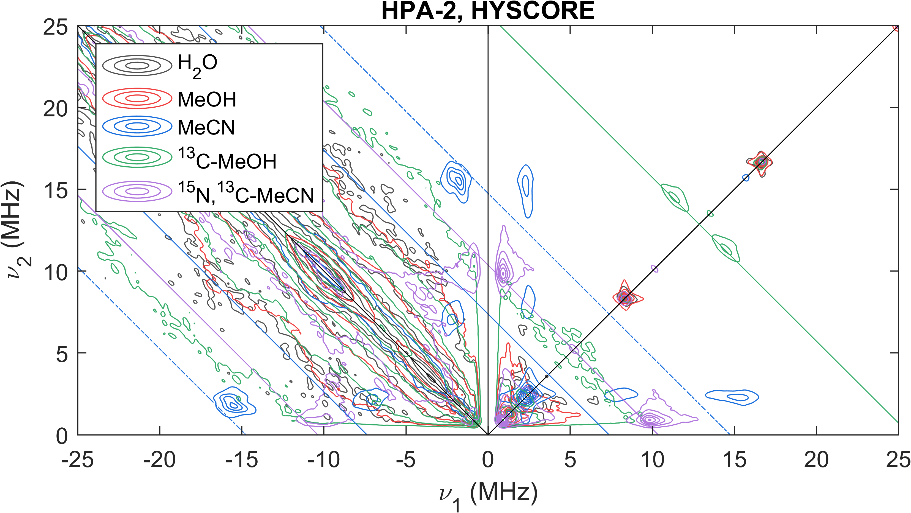 |
| --- | --- |

*Figure S23: HYSCORE spectra of HPA-2 starting solutions in the different environments used in this work. Left: full spectra (ν_max_ = 62.5 MHz); right: frequency axes limited to 25 MHz.*

### HYSCORE experiments on the H_2_O:MeCN samples

The effect of *τ*-dependent blind spotting on the 2D frequency spectra was checked on one sample, namely the catalyst in H_2_O:MeCN before the reaction; the results, shown in Figure S24 confirm that the blind spots do not affect the peaks of interest.

| 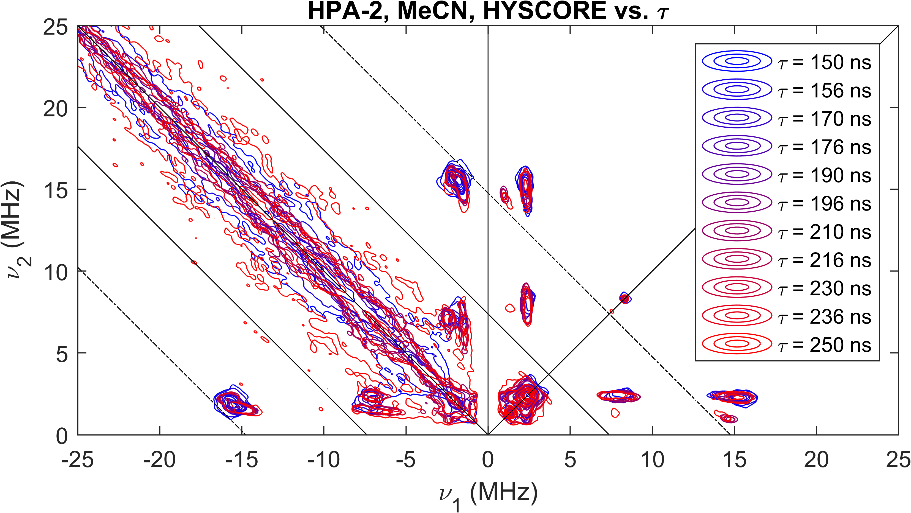 | 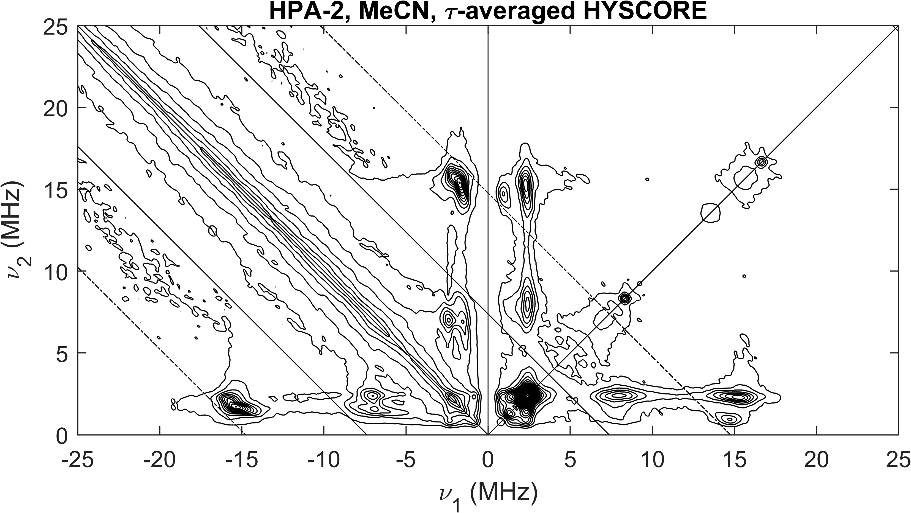 |
| --- | --- |

*Figure S24: HPA-2 in H_2_O:MeCN. Left: τ-dependent 4-pulse HYSCORE spectra. Right: τ-averaged 4-pulse HYSCORE spectrum.*

The cross-peaks visible in Figure S24 could be conclusively assigned to the hyperfine with the ^14^N nucleus of the solvent additive (*I* = 1, 99.64% nuclear abundance) by performing an experiment in the presence of ^15^N,^13^C-labelled MeCN (see Figure S25), causing the peaks to shift as a result of the isotope substitution (^14^N: *I* = 1, γ_N_/γ_N_(^1^H) = 0.072; ^15^N: *I* = 1/2, γ_N_/γ_N_(^1^H) = -0.101). No clear signals could be attributed to the coupling with the ^13^C nucleus of ^15^N,^13^C-labelled MeCN (*I* = 1/2, γ_N_/γ_N_(^1^H) = 0.252).


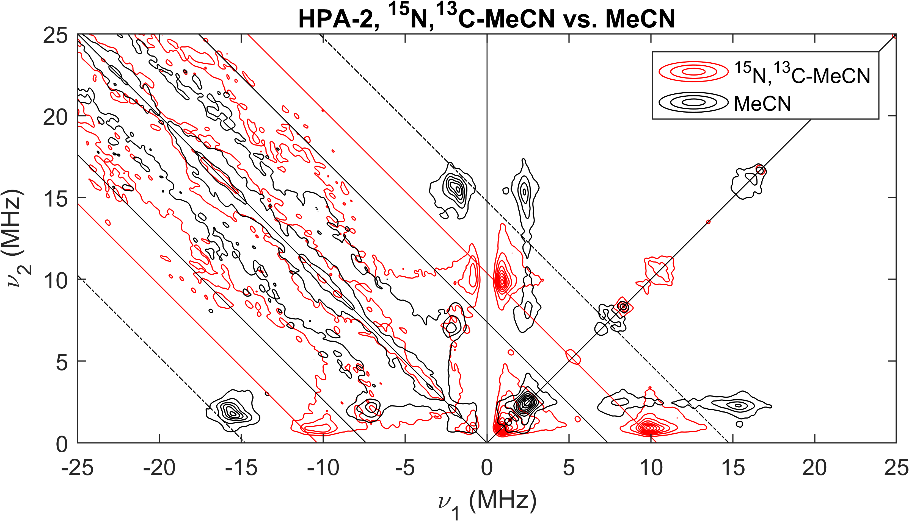


*Figure S25: HPA-2 in H_2_O:MeCN (black) vs. H_2_O:^15^N,^13^C-MeCN (red). For both experiments a τ of 156 ns was used.*

To confirm the position of the resonances, the full set of ^14^N cross-peaks, including correlations within the same electron spin manifold, was obtained by performing a Double Nuclear Coherence Transfer (DONUT)-HYSCORE^8^ experiment (see Figure S26). This is based on the 5-pulse sequence

(π/2) – τ – (π/2) – *t*_1_ – (π) – T_mix_ – (π) – *t*_2_ – (π/2) – τ – echo

in which the echo intensity is recorded as a function of the time delays *t*_1_ and *t*_2_ whereas the time intervals τ and T_mix_ are kept constant.

The time intervals *t*_1_ and *t*_2_ were varied independently starting from 80 ns in steps of 8 ns each; 256 points were collected along each time dimension, corresponding to maximum *t*_1_ and *t*_2_ values of 2.120 µs. In analogy with the corresponding HYSCORE experiment, *τ* was set to 156 ns. T_mix_ was set to 400 ns; this value was optimised by monitoring the signal of the additional peaks in a series of 1D spectra recorded as a function of T_mix_ in which *t*_1_ and *t*_2_ were incremented simultaneously. The length of the π/2 and π pulses was set to 6 ns and 12 ns, respectively; the echo was integrated over a 10 ns gate. A 16-step phase cycle x(x)(x)(x), built upon the 8-step phase cycle for HYSCORE x(x)(x)(x)^4^ with the addition of a 2-step phase cycle on the second π pulse of the mixing block,^9^ was used to select the coherence transfer pathways yielding the desired signal. Data acquisition took approximately 12 hours; the time-domain signal was processed following the same procedure outlined for the HYSCORE spectra.


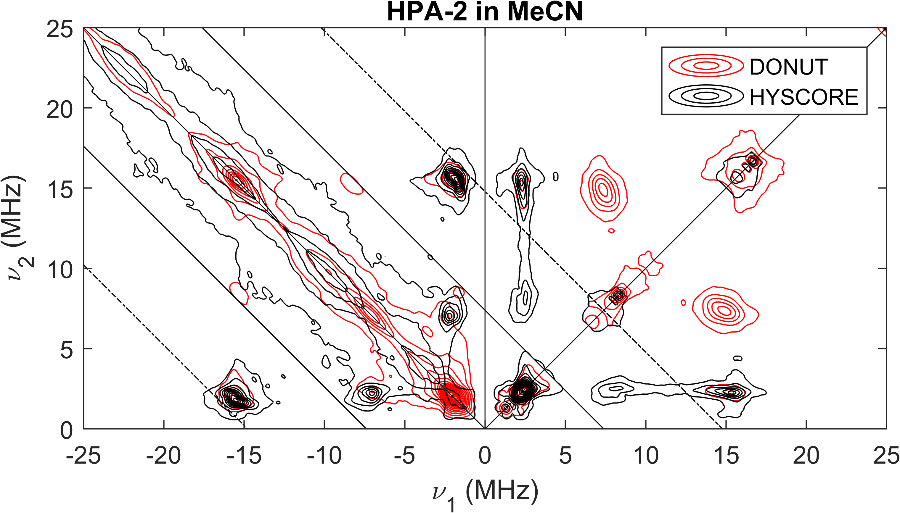


*Figure S26: HYSCORE (black) and DONUT-HYSCORE (red) spectra of HPA-2 in H_2_O:MeCN. HYSCORE: τ = 156 ns; DONUT-HYSCORE: τ = 156 ns, T_mix_ = 400 ns.*

The HYSCORE spectra in the presence of MeCN or ^15^N,^13^C-MeCN as the solvent additive could be simulated by considering the coupling with a single Nitrogen nucleus (see Figure S27).

| 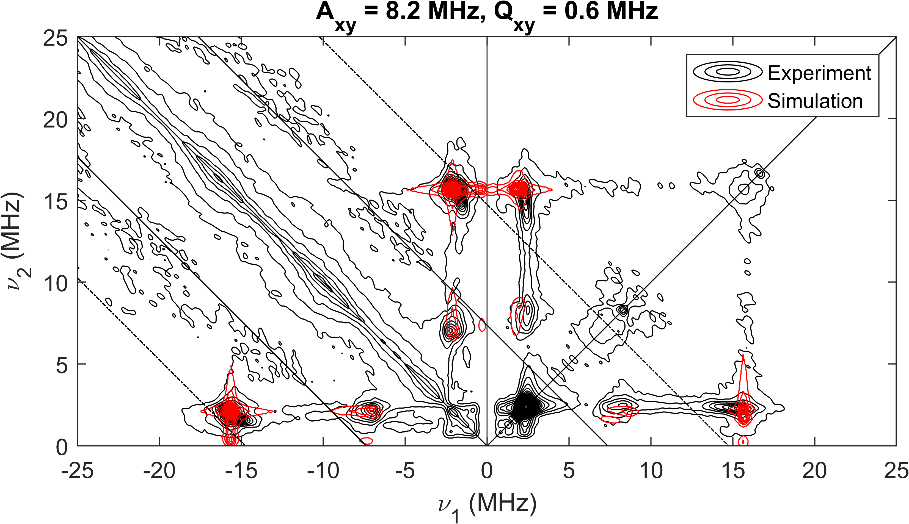 | 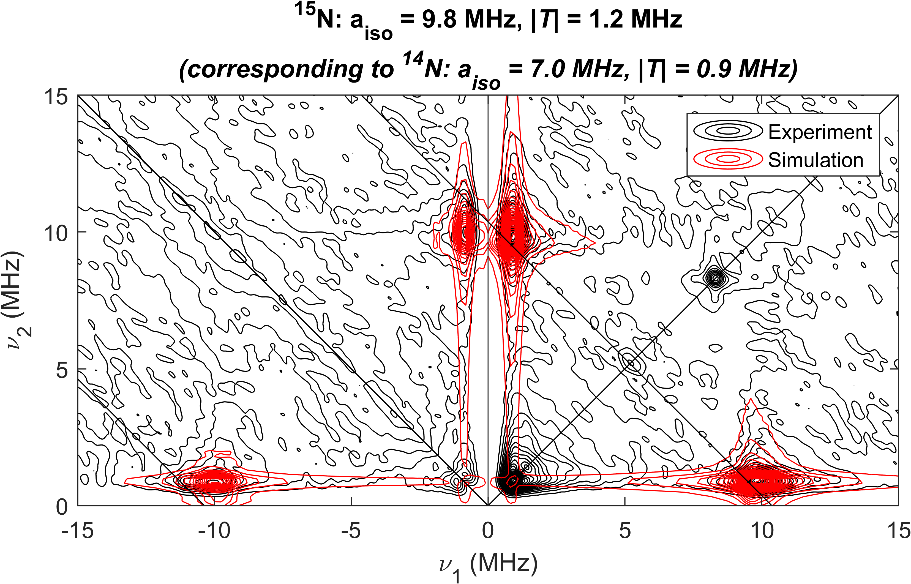 |
| --- | --- |

*Figure S27: Experimental (black) and simulated (red) HYSCORE spectra of HPA-2 in the presence of MeCN (left) or ^15^N,^13^C-MeCN (red) as the solvent additive.*

The set of peaks arising from the coupling with the ^14^N nucleus of MeCN (*I* = 1) could be satisfactorily reproduced assuming an effective value of the hyperfine coupling *A* of 8.2 MHz and a quadrupolar splitting *Q* of 0.6 MHz (see Figure S27 left). Due to the orientational selection imposed by the choice of the excitation position with respect to the EPR spectrum of the vanadyl moiety (see Fig. S24 right), the obtained hyperfine and quadrupolar coupling values are a projection of the corresponding interaction matrices over the *xy* plane defined by the axial symmetry of the electron Zeeman (***g***) and Vanadium hyperfine (^V^***A***) interactions.

Thanks to the absence of the nuclear quadrupolar interaction, only relevant for nuclei with *I* ≥ 1 due to their non-spherical charge distribution, the spectrum obtained upon isotope labelling of MeCN with ^15^N (*I* = 1/2) enables the separate determination of the isotropic (*a*_iso_, mostly through-bond) and dipolar (*T*, through-space) components of the hyperfine coupling, related to the full hyperfine matrix ***A*** by ***A*** = *a*_iso_·[1 1 1] + *T*·[-1 -1 +2] assuming an axial symmetry.

The starting point for the simulation was determined by displaying the HYSCORE data in a ν_1_² *vs.* ν_2_² plot (see Figure S28); in such a representation, the coordinates of the cross peaks fall onto straight lines^10^ $\nu_{2}^{2}=Q\nu_{1}^{2}+G$ with $|a_{iso}+\frac{T}{2}|=|2\nu_{N}\frac{1+Q}{1-Q}|$ and $T^{2}=\frac{16}{9(1-Q)}(G+\frac{4\nu_{N}^{2}Q}{1-Q})$ where $\nu_{N}$ is the Larmor frequency of the nucleus coupled to the unpaired electron spin.^11^


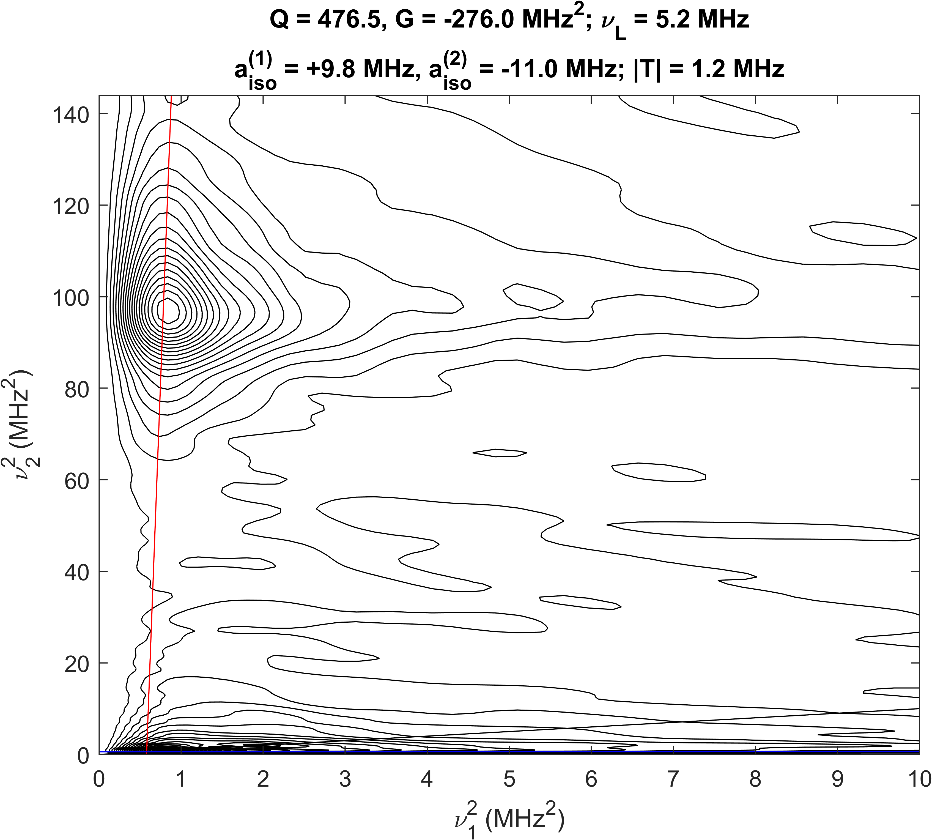


*Figure S28: ν_1_² vs. ν_2_² plot corresponding to Figure S27 right. The values of Q and G correspond to the displayed fitting lines.*

Solving for *a*_iso_ and *T* from the values of *Q* and *G* determined from the analysis of the experimental data yields two distinct solutions; the correct set of coupling parameters can be determined by comparing the simulated spectrum with the experimental one (see Figure S24 right).

Within the framework of the point-dipole approximation it is possible to estimate the distance between the unpaired electron and the coupled nucleus from the magnitude of the dipolar part *T* of the hyperfine interaction as $r=\sqrt[3]{\frac{\mu_{0}}{4\pi h}\frac{g_{e}g_{n}\mu_{B}\mu_{N}}{T_{\perp}}}$ in which *μ*_0_ is the vacuum permeability, *g_e_* is the *g* value of the electron spin, *g_n_* is the *g* value of the nuclear spin, *μ_B_* is the Bohr magneton, *μ_N_* is the nuclear magneton and *T*_⊥_ is the in-plane component of the dipolar part of the hyperfine coupling interaction in its reference frame (***T*** = [-T_⊥_ -T_⊥_ 2T_⊥_]). For a ^15^N nucleus the distance corresponding to *T*_⊥_ = 1.2 MHz is ~1.9 Å.

The position of the peaks in the HYSCORE spectra does not change across the catalysis experiment (see Figure S29), suggesting a stable coordination geometry of the solvent additive.

| 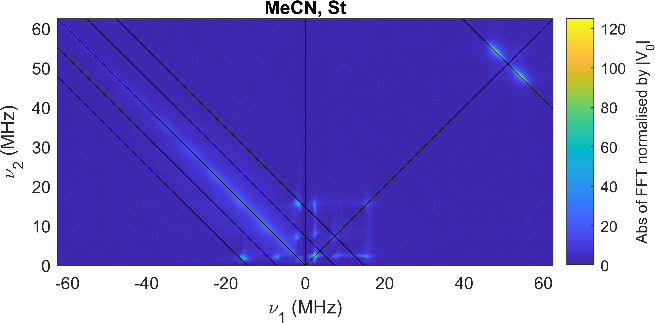 | 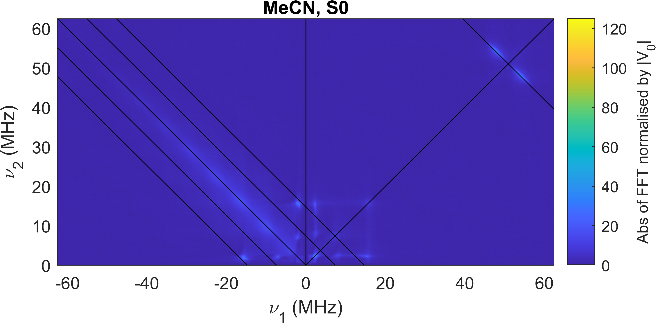 | 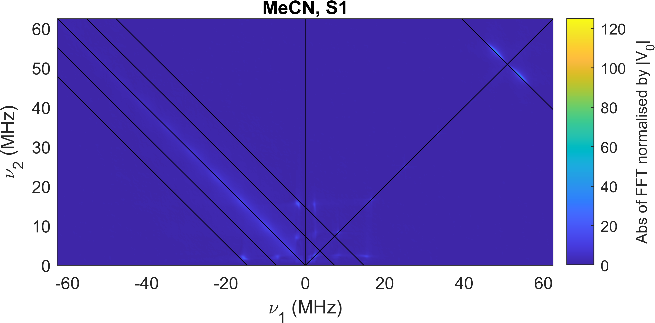 |
| --- | --- | --- |
| 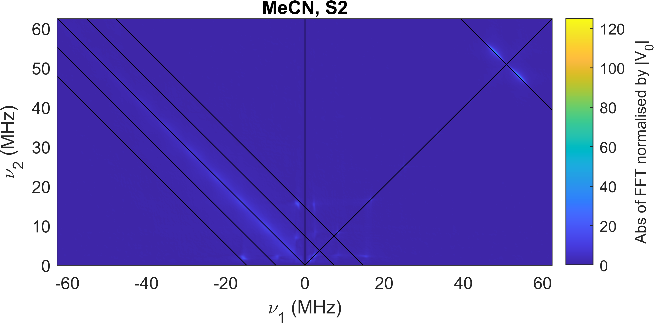 | 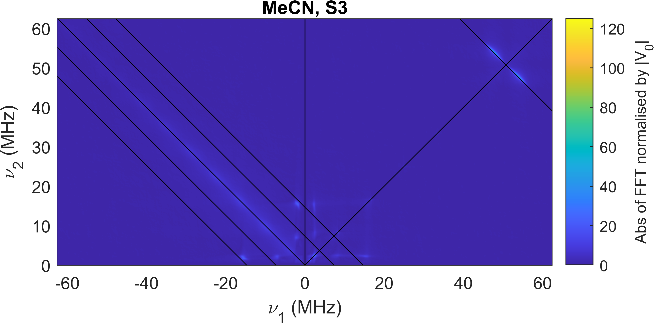 | 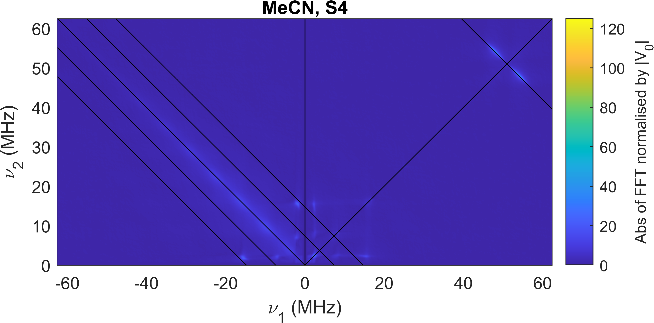 |
| 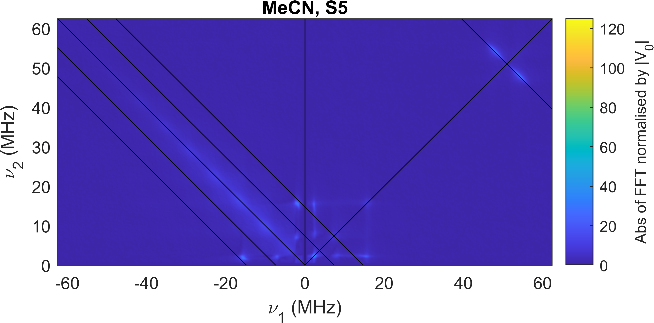 | 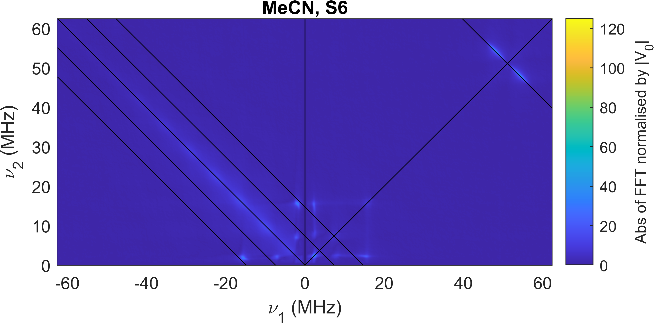 | 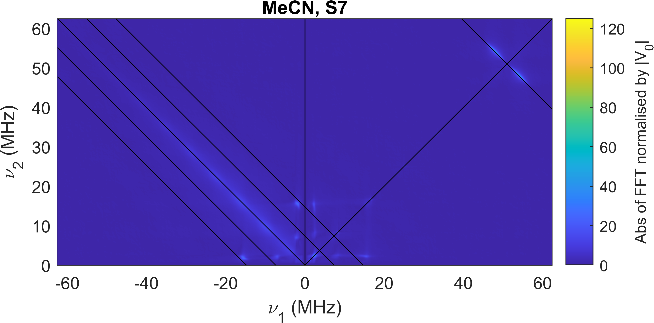 |
| 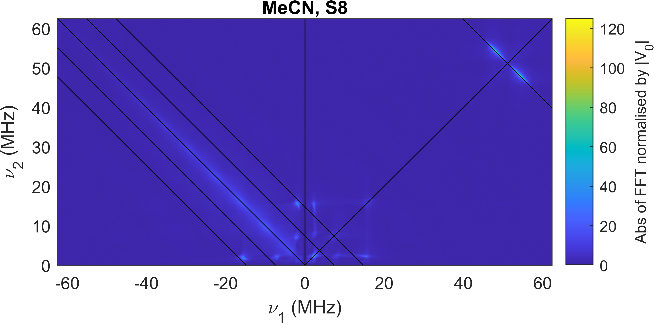 | 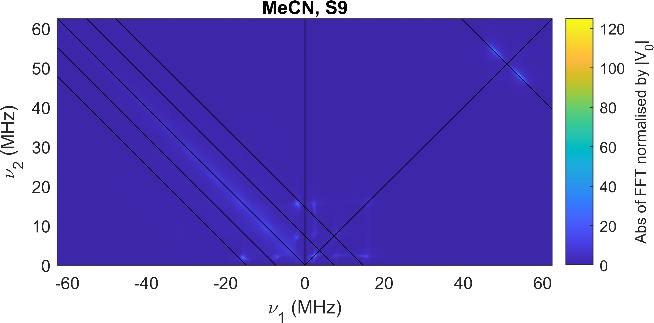 | 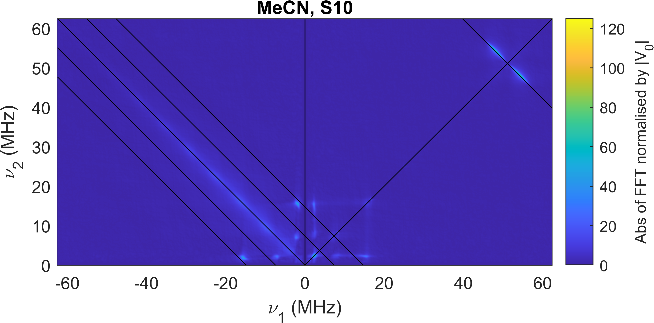 |

*Figure S29: HYSCORE spectra of HPA-2 in H_2_O:MeCN for the different samples collected along the reaction coordinate. To enable the comparison of the peak intensities, all the spectra have been normalised by the absolute value of the starting echo signal (t_1_ = t_2_ = 80 ns); the colour scale is the same throughout the series of plots.*

HYSCORE experiments on the H_2_O:MeOH samples

A sample with ^13^C-MeOH as additive has been prepared to characterise the interaction of the MeOH solvent additive with the HPA-2 catalyst by probing the interaction with the NMR-active ^13^C nucleus (*I* = 1/2, γ_N_/γ_N_(^1^H) = 0.252).

The HYSCORE spectra, displayed in Figure S30, show a weak set of signals in the (+,+) quadrant that could be conclusively attributed to the hyperfine interaction with the ^13^C nucleus of the isotopically labelled solvent additive by comparison with the spectrum of the corresponding unlabelled sample. The position and shape of the peaks are not affected by the τ-dependent blind spots of the 4-pulse HYSCORE experiments (see Figure S30 right).

| 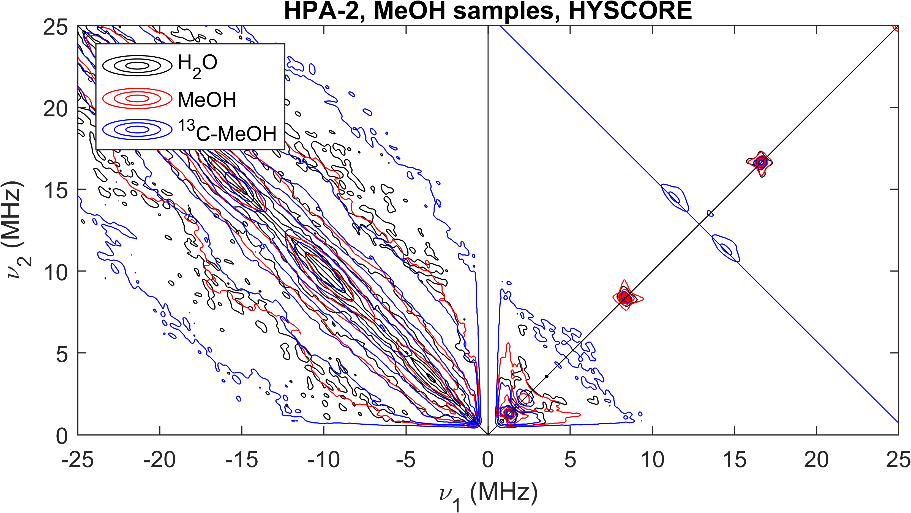 | *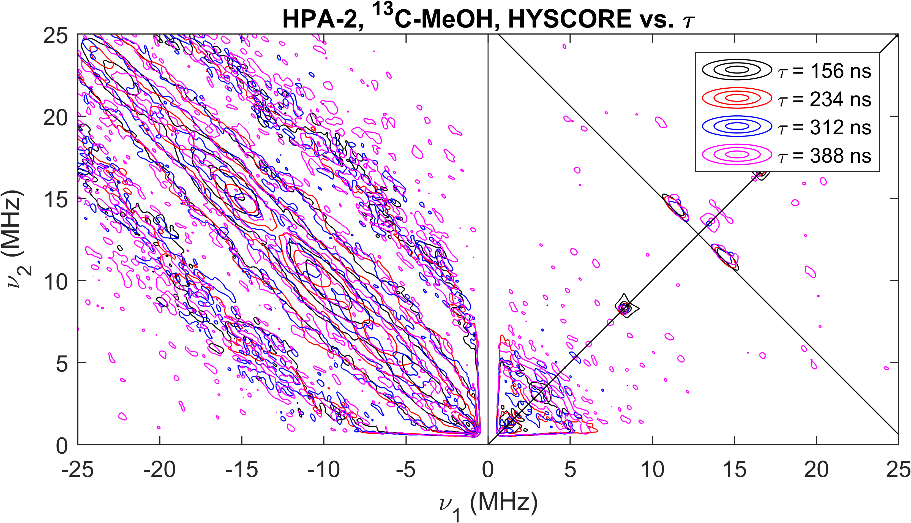* |
| --- | --- |

*Figure S30: HYSCORE spectra of HPA-2 in H_2_O:^13^C-MeOH. Left: comparison between H_2_O:MeOH and H_2_O:^13^C-MeOH, confirming that the cross-peaks can be attributed to the interaction with the NMR-active ^13^C nucleus; the displayed spectrum for the labelled sample was obtained as the sum of the traces recorded with τ = 156 ns, 234 ns, 312 ns and 388 ns whereas the one for the unlabelled sample was obtained with a single τ value of 156 ns. Right: τ-dependent 4-pulse HYSCORE spectra of HPA-2 in H_2_O:^13^C-MeOH.*

As ^13^C has a nuclear spin *I* = 1/2, the hyperfine coupling parameters can be extracted from a ν_1_² *vs.* ν_2_² plot following the procedure highlighted above for the interaction with ^15^N (see Figure S31).

| 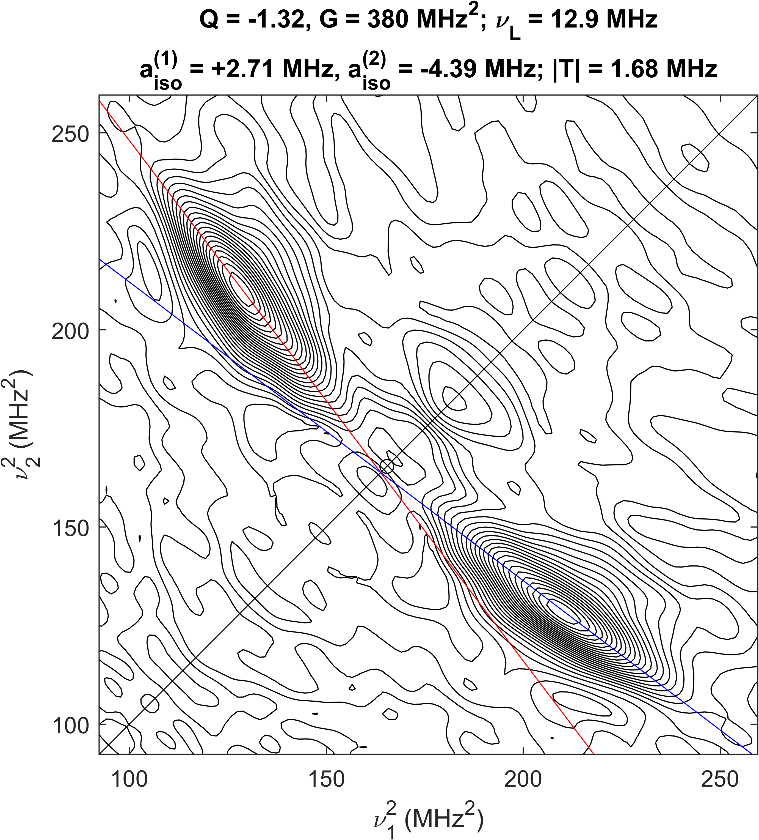 |  | 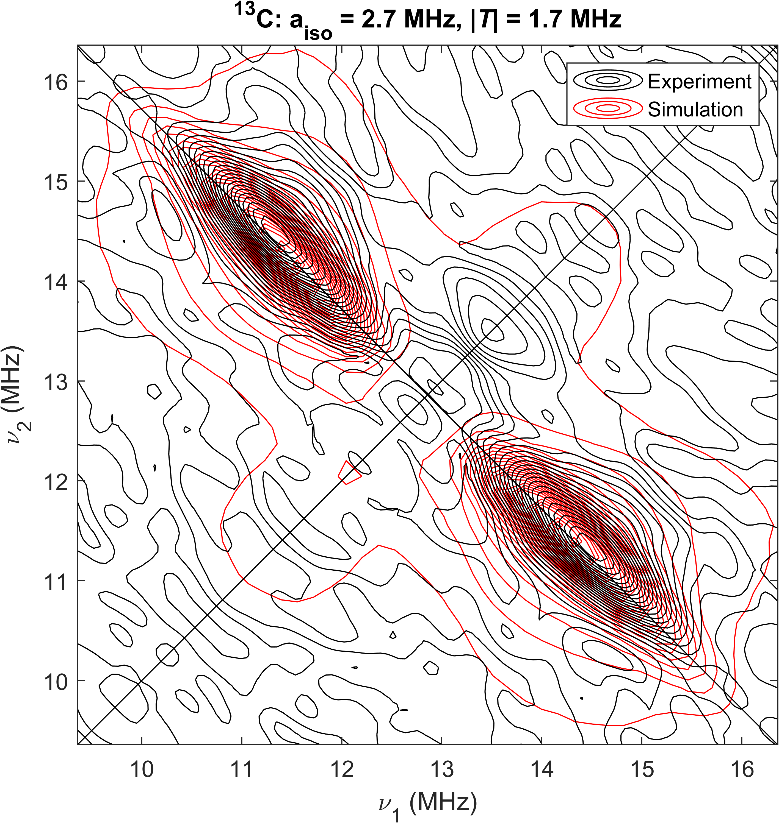 |
| --- | --- | --- |

*Figure S31 Left: ν_1_² vs. ν_2_² plot corresponding to the blue trace in Figure S33 left; the values of Q and G correspond to the displayed fitting lines. Right: simulation of the ^13^C cross-peaks of HPA-2 in H2O:^13^C-MeOH.*

The obtained value for the dipolar part of the ^13^C hyperfine interaction, *T* = 1.7 MHz, corresponds to a distance between the unpaired electron and the ^13^C nucleus of ~2.3 Å within the framework of the point-dipole approximation (see the paragraph above on *HYSCORE experiments on the H_2_O:MeCN samples* for further details).

## 4.7 Additional results of spectroscopic measurements of reaction samples

| H_2_O |  |
| --- | --- |
| H_2_O:MeOH=9:1 |  |
| H_2_O:MeCN=9:1 |  |

Figure S32: ^51^V-NMR spectra of reaction solutions for investigations of catalyst-solvent interactions. Blue arrow shows sample order increasing with reaction time.

# **5. References**

(1) Huber, M.; Poller, M. J.; Tochtermann, J.; Korth, W.; Jess, A.; Albert, J. Revealing the Nitrogen Reaction Pathway for the Catalytic Oxidative Denitrification of Fuels. *Chemical Communications* **2023**, *59* (27), 4079–4082. https://doi.org/10.1039/d3cc00648d.

(2) Lin, G.; Liu, S.; Shi, D.; Yang, Y.; Yu, F.; Lu, T.; Yu, X.-Y.; Zhao, Y. A New [PMo12O40]3−-Based NiII Compound: Electrochemical and Photocatalytic Properties for Water Pollutant Removal. *Molecules* **2025**, *30* (10), 2172. https://doi.org/10.3390/molecules30102172.

(3) Huang, B.-Q.; Wang, L.; Shi, K.; Xie, Z.-X.; Zheng, L.-S. A New Strategy for the Fabrication of the Phosphor Polyoxomolybdate Modified Electrode from Ionic Liquid Solutions and Its Electrocatalytic Activities. *Journal of Electroanalytical Chemistry* **2008**, *615* (1), 19–24. https://doi.org/10.1016/j.jelechem.2007.11.022.

(4) Stoll, S.; Schweiger, A. EasySpin, a Comprehensive Software Package for Spectral Simulation and Analysis in EPR. *Journal of Magnetic Resonance* **2006**, *178* (1), 42–55. https://doi.org/10.1016/j.jmr.2005.08.013.

(5) Stoll, S.; Kasumaj, B. Phase Cycling in Electron Spin Echo Envelope Modulation. *Applied Magnetic Resonance* **2008**, *35* (1), 15–32. https://doi.org/10.1007/s00723-008-0140-6.

(6) Höfer, P.; Grupp, A.; Nebenführ, H.; Mehring, M. Hyperfine Sublevel Correlation (Hyscore) Spectroscopy: A 2D ESR Investigation of the Squaric Acid Radical. *Chemical Physics Letters* **1986**, *132* (3), 279–282. https://doi.org/10.1016/0009-2614(86)80124-5.

(7) Fábregas Ibáñez, L.; Soetbeer, J.; Klose, D.; Tinzl, M.; Hilvert, D.; Jeschke, G. Non-Uniform HYSCORE: Measurement, Processing and Analysis with Hyscorean. *Journal of Magnetic Resonance* **2019**, *307*. https://doi.org/10.1016/j.jmr.2019.106576.

(8) Goldfarb, D.; Kofman, V.; Libman, J.; Shanzer, A.; Rahmatouline, R.; Van Doorslaer, S.; Schweiger, A. Double Nuclear Coherence Transfer (DONUT)-HYSCORE: A New Tool for the Assignment of Nuclear Frequencies in Pulsed EPr Experiments. *Journal of the American Chemical Society* **1998**, *120* (28), 7020–7029. https://doi.org/10.1021/ja973271r.

(9) Pöppl, A.; Böttcher, R.; Völkel, G. Analysis of Anisotropic Hyperfine Interaction Distributions in Two-Dimensional Four- and Five-Pulse Electron Spin Echo Envelope Modulation Spectra of Protons in Single Crystals. *Applied Magnetic Resonance* **1997**, *12* (1), 15–39. https://doi.org/10.1007/BF03161988.

(10) Dikanov, S. A.; Bowman, M. K. Cross-Peak Lineshape of Two-Dimensional ESEEM Spectra in Disordered S = 1 2, I = 1 2 Spin Systems. *Journal of Magnetic Resonance, Series A*. 1995, pp 125–128. https://doi.org/10.1006/jmra.1995.1199.

(11) Savitsky, A.; Nalepa, A.; Petrenko, T.; Plato, M.; Möbius, K.; Lubitz, W. Hydrogen-Bonded Complexes of Neutral Nitroxide Radicals with 2-Propanol Studied by Multifrequency EPR/ENDOR. *Applied Magnetic Resonance* **2022**, *53* (7–9), 1239–1263. https://doi.org/10.1007/s00723-021-01442-y.
